# Agro-physiological responses and molecular docking evaluation of some essential oils and ascorbic acid as eco-friendly inhibitors of powdery mildew in cucumber plants

**DOI:** 10.3389/fpls.2026.1786347

**Published:** 2026-06-12

**Authors:** Hanaa S. Omar, Ahmed Mohamed, Nehad A. El-Gammal, Reda I. Omara, Amira A. Ayad, Mohamed M. El-Mogy, Leonard L. Williams, Emad A. Abdeldaym

**Affiliations:** 1 Genetics, Faculty of Agriculture, Cairo University, Giza, Egypt; 2 Plant Pathology Research Institute, Agriculture Research Center, Giza, Egypt; 3 Center for Excellence in Post-Harvest Technologies, North Carolina Agricultural and Technical State University, Kannapolis, NC, United States; 4 Department of Arid Land Agriculture, College of Agricultural and Food Sciences, King Faisal University, El-Mogy, Saudi Arabia; 5 Department of Vegetable, Faculty of Agriculture, Cairo University, Giza, Egypt

**Keywords:** antioxidant enzymes, *Cucumis sativus*, essential oils, molecular docking, powdery mildew, resistance induction

## Abstract

**Introduction:**

Powdery mildew is a major fungal disease affecting cucumber production, often managed using synthetic fungicides with environmental and health concerns. This study investigates plant-derived essential oils and ascorbic acid as eco-friendly alternatives, focusing on their effects on disease control, plant physiology, biochemical defenses, and molecular resistance mechanisms.

**Methods:**

Two greenhouse experiments were conducted using garlic oil, cumin oil, ascorbic acid, a commercial fungicide (Topas), and an untreated control. Antifungal activity was evaluated alongside plant growth, physiological traits, and yield parameters. Oxidative stress markers (H₂O₂ and MDA), antioxidant enzyme activities (PPO and POD), and biochemical compounds (TPC, TFC, TFAA, and proline) were measured. Gene expression of defense-related markers (PR-1 and LOX-1) was analyzed using qRT-PCR. Gas chromatography–mass spectrometry (GC-MS) identified major bioactive compounds, and molecular docking was performed to explore potential antifungal mechanisms.

**Results:**

All treatments significantly reduced powdery mildew severity and oxidative stress compared with untreated plants. Garlic oil showed the highest efficacy, followed by cumin oil and ascorbic acid. Disease severity was positively correlated with H₂O₂ and MDA levels and negatively correlated with plant growth, chlorophyll content, and yield. Garlic oil significantly enhanced plant height, biomass, leaf number, chlorophyll content, and fruit yield. These improvements were associated with increased antioxidant enzyme activities and higher levels of phenolics, flavonoids, amino acids, and proline. Gene expression analysis revealed significant upregulation of PR-1 and LOX-1, particularly in garlic and cumin oil treatments. Molecular docking supported the antifungal potential of the identified compounds through stable interactions with key fungal targets.

**Discussion:**

Plant-derived essential oils, especially garlic oil, demonstrated strong antifungal activity and the ability to enhance plant defense systems at physiological, biochemical, and molecular levels. These findings highlight their potential as safe, sustainable alternatives to synthetic fungicides for managing powdery mildew in cucumber cultivation.

## 1 Introduction

Cucumber (*Cucumis sativus* L.) is one of the most economically important vegetable crops within the Cucurbitaceae family, with its value reflected in both domestic consumption and export markets worldwide (El-Mogy et al., 2025). Cucumber cultivation is widely practiced under open-field and protected conditions, particularly in greenhouse systems, which are increasingly adopted to extend production seasons, enhance yield stability, and protect crops from adverse environmental conditions, pests, and diseases (Kaur and Sharma, 2022). However, protected cultivation often creates microclimatic conditions—such as high humidity and moderate temperatures—that favor the development and rapid spread of foliar fungal diseases.

Among these diseases, powdery mildew is considered one of the most destructive constraints to cucumber production globally (Aroge et al., 2025). The disease affects all aerial parts of the plant, particularly leaves, leading to reduced photosynthetic efficiency, premature senescence, and substantial yield and quality losses (Mostafa et al., 2021). In Egypt and many other cucumber-producing regions, powdery mildew is primarily caused by *Podosphaera xanthii*, which has been identified as the dominant causal agent rather than *Erysiphe cichoracearum* (El-Kazzaz, 1981; Elagamey et al., 2023). Typical symptoms include the appearance of white powdery fungal colonies on leaf surfaces and stems, which progressively expand and severely impair plant physiological performance (Ji et al., 2024). Infection by *P. xanthii* disrupts photosynthetic activity, accelerates fruit maturation, and ultimately results in significant economic losses (Mieslerová et al., 2022; Aroge et al., 2025).

Management of cucumber powdery mildew traditionally relies on frequent applications of synthetic fungicides. Although chemical fungicides can provide rapid disease suppression, their intensive and repeated use has led to several critical challenges. These include the development of fungicide-resistant pathogen populations, reduced long-term efficacy, contamination of agricultural ecosystems, and growing concerns regarding food safety and human health (Vielba-Fernández et al., 2020; Leonardi et al., 2023). Moreover, resistance of *P. xanthii* to commonly used fungicides has been reported, further limiting the effectiveness of chemical control strategies (McGrath, 1996; McGrath and Shishkoff, 1999).Consequently, there is an urgent need to develop alternative fungal disease management approaches that are effective, environmentally safe, and compatible with sustainable agriculture (Sharma et al., 2024; Abdelfatah et al., 2025). In this context, plant-derived natural products, particularly essential oils, have received increasing attention as promising alternatives to synthetic fungicides. Essential oils are complex mixtures of bioactive secondary metabolites, including terpenes, alcohols, aldehydes, ketones, and phenolic compounds, which exhibit a wide range of biological activities such as antimicrobial, antifungal, insecticidal, and herbicidal effects (Lee et al., 2019; Gupta et al., 2023). Due to their natural origin, rapid biodegradability, and relatively low toxicity to non-target organisms, essential oils are considered low-risk substances and represent an important component of integrated pest management (IPM) strategies (Martini et al., 2023).

Several studies have demonstrated the efficacy of essential oils in suppressing foliar fungal diseases, including powdery mildew, leaf spots, and blights, in various horticultural crops (Parikh et al., 2021; Mostafa et al., 2021). Garlic (*Allium sativum*) and cumin (*Cuminum cyminum*) oils are particularly rich in bioactive compounds, such as sulfur-containing constituents and aromatic aldehydes, which exhibit strong antifungal activity. These compounds inhibit fungal growth by disrupting cell membrane integrity and interfering with essential metabolic processes in plant pathogens. Moreover, essential oils are generally regarded as safe for mammals, supporting their potential use in environmentally friendly disease management strategies (Chaudhari et al., 2021). However, despite their well-documented antimicrobial activity under laboratory conditions, their field application remains limited due to lower persistence and variable efficacy compared with synthetic fungicides (Bolouri et al., 2022). Therefore, comprehensive evaluations under controlled and semi-commercial conditions are necessary to determine their practical effectiveness, optimal application rates, and potential effects on plant growth and productivity.

In addition to essential oils, ascorbic acid (vitamin C) has been reported to play a role in enhancing plant defense responses. Ascorbic acid functions as a key antioxidant involved in scavenging reactive oxygen species (ROS), maintaining cellular redox balance, and modulating plant immunity (Biswas et al., 2020; Noor and Little, 2022). Exogenous application of ascorbic acid has been shown to improve plant tolerance to biotic stress by reducing oxidative damage and stimulating defense-related biochemical pathways (Conklin et al., 2024). However, its combined evaluation with essential oils for managing cucumber powdery mildew remains insufficiently explored. Beyond conventional biological assays, advances in computational biology provide valuable tools for understanding plant–pathogen–compound interactions at the molecular level (Morris et al., 2009). Molecular docking analysis has emerged as a powerful approach to predict the binding affinity and interaction mechanisms between bioactive compounds and target proteins (Trott and Olson, 2010; Forli et al., 2016). In fungal pathogens, cell wall biosynthesis proteins are critical for growth, structural integrity, and pathogenicity (Brain et al., 2025). In powdery mildew pathogens, chitin synthase (CHS) is an essential enzyme in *Podosphaera xanthii*, responsible for the synthesis of chitin required for the construction and maintenance of the fungal cell wall, particularly during haustorial development involved in cucumber infection. This makes CHS an attractive target for antifungal compounds (Bakhat et al., 2023). To date, no studies have explored the potential modes of action of garlic and cumin oil constituents against *P. xanthii* virulence proteins using molecular docking approaches.”

Therefore, the present study aimed to (i) identify and characterize the bioactive chemical constituents of garlic and cumin essential oils using gas chromatography-mass spectrometry (GC–MS); (ii) evaluate the efficacy of garlic oil, cumin oil, and ascorbic acid in comparison with the commercial fungicide Topas for controlling cucumber powdery mildew under greenhouse conditions over two consecutive growing seasons; (iii) assess their effects on disease severity, plant growth, physiological performance, and yield; and (iv) elucidate the potential antifungal mechanisms of key essential oil components through molecular docking analysis targeting *P. xanthii* cellulose synthase-related proteins. This integrated approach provides both experimental and molecular insights into the feasibility of using plant-derived natural compounds as sustainable alternatives to synthetic fungicides in cucumber disease management.

## 2 Materials and methods

### 2.1 Disease diagnosis and visual assessment

In current study, natural infection of powdery mildew induced by *P. xanthii* was relied upon within the greenhouse conditions. The experiment was performed in a greenhouse with a known history of recurrent powdery mildew infection. This is ensuring the presence of sufficient primary inoculum derived from conidia remaining on plant debris, support wires, and internal greenhouse structures. During the experimental period, cucumber plants were allowed to grow under normal greenhouse conditions without the application of any preventive treatments. This is permitting infection to occur naturally and to reflect real production conditions. The prevailing environmental conditions in the greenhouse—moderate temperatures (24 ± 2 °C) and relatively high humidity(70 ± 4%)—favored spore dispersal and disease initiation. Disease development was monitored at regular intervals beginning with the appearance of the initial symptoms, characterized by fine white powdery spots on the upper leaf surface, to assess the progression of infection and the response of cucumber plants to the applied treatments. The characteristic symptoms of the pathogen, including white powdery patches on leaf surfaces and conidial spores of P. xanthii, were documented and are presented in **Supplementary Figure 1**.

### 2.2 Essential oil preparation and treatment applications

Essential oils of garlic (Allium sativum L.) and cumin (Cuminum cyminum L.) were procured from El-Captain Company (Cap Pharm, Cairo, Egypt). For foliar applications, the oils were prepared at a concentration of 3000 ppm, previously determined as the optimal dose based on preliminary inhibitory screening. Due to the hydrophobic nature of essential oils, emulsions were formulated using Arabic gum as a stabilizer at 0.5 g L^−1^ (w/v), a concentration optimized to ensure emulsion stability without affecting biological activity. Emulsions were prepared by high-speed mechanical stirring followed by magnetic homogenization for 20 min to achieve uniform dispersion. A stock solution was first prepared and subsequently diluted to the required working concentrations. All treatments were freshly prepared before each application and vortexed briefly prior to use to prevent phase separation.

The experimental design included two control groups: (i) a chemical control using the commercial fungicide Topas^®^ EC 100 (10% penconazole, Syngenta, Egypt) applied at 25 mL per 100 L, and (ii) a negative (vehicle) control consisting of sterile distilled water supplemented with Arabic gum (0.5 g L^−1^) to verify that the emulsifier exerted no independent effect on the pathogen. Treatments were initiated at the first appearance of powdery mildew symptoms and applied three times at 10-day intervals. Disease severity was recorded every 10 days for a total of four assessments, with the final evaluation conducted one week after the last application. Disease development was quantified using the Area Under the Disease Progress Curve (AUDPC).

To minimize potential interference from essential oil residues and formulation components in subsequent biochemical analyzes, plant extracts were centrifuged at 10,000 rpm for 15 min prior to analysis. In addition, sample blanks containing assay reagents and essential oils without plant extract were used to subtract background absorbance and fluorescence signals. Further controls were implemented to account for possible interference from essential oil constituents and the emulsifier (Arabic gum). A vehicle control consisting of plants treated with aqueous Arabic gum solution (0.5 g L^−1^) without essential oils was included to evaluate any independent effect of the emulsifier. Moreover, for each spectrophotometric or fluorometric assay, a matrix blank containing all assay reagents together with Arabic gum (0.5 g L^−1^) and essential oils at the working concentration (3000 ppm), but without plant tissue, was prepared and processed identically to the tested samples. The absorbance or fluorescence values obtained from these blanks were subtracted from the corresponding sample readings to correct for background optical interference arising from formulation components. This procedure ensured that the recorded measurements accurately reflected the biological responses of plant tissues rather than formulation-related artifacts.

### 2.3 Greenhouse experiment

#### 2.3.1 Plant materials, greenhouse conditions, and experimental design

Cucumber plants (Barracuda cucumber variety from Suez Canal Company) are the most susceptible plants for powdery mildew disease. The seeds were surface sterilized with sodiumhypochlorite (1.5%) for 15 min, rinsed three times with distilled water, and then sown in cell trays filled with a mixture of peat moss, perlite, and vermiculite (1:1:1). After 15 days old, the seedlings were transplanted into plastic greenhouse at horticulture institute, Agriculture Research Centre, Giza, Egypt, during the two spring seasons of 2023 and 2024.Seedlings transplanted in controlled greenhouse at a spacing of 50 cm using a double row in each ridge. The plot was 3.5 m in length and 1.5 m in width; each plot had 14 plants. The treatments were arranged in a randomized complete block design with four replicates. The plants were irrigated regularly for two weeks and fertilized with mineral nutrients at rates of 50 kg ammonium sulfate, 25 kg urea, and 60 kg potassium sulfate per feddan. The cucumber plants were exposed to the natural infection by *P. xanthii.*

### 2.4 Disease assessment

#### 2.4.1 Disease severity

The severity of powdery mildew disease on cucumber leaves was evaluated using a scale containing five categories established by Morishita et al. (2003): 0 = No visible infection lesions; 1 = 25% or less of leaf area infected; 2 = 26-50% of leaf area infected; 3 = 51–75 of leaf area infected; and 4 = 76-100% of leaf area infected. The ratio of disease severity was assessed using the following equation:


$$\text{Ratio of disease severity}\left(\text{DS}\right)=\frac{\sum \left(\text{n}\times \text{c}\right)}{\text{N}\times \text{C}}\times 100$$

Whereas: n = Number of infected leaves per category, c = Category number, N = Total examined leaves, C = the highest category number of infection.

#### 2.4.2 The area under disease progress curve

##### 2.4.2.1 Area under disease progress curve

The area under the disease progress curve (AUDPC) was estimated to compare the responses of different tested cultivars and to more accurately characterize partial resistance. It was calculated according to Pandey et al. (1989) using the following equation:


AUDPC=D[12(Y1+YK)+Y2+Y3+…+Y(K−1)]

Where:

D = Intervals of time (days between recordings of consecutive diseases).

Y1 + Yk = The total of the initial and final disease scores.

Y2 + Y3 + …. + Y (K−1) = Sum of all in between disease scores.

### 2.5 Plant growth parameters and fruit yield

Five cucumber plants were randomly selected from the middle section of each plot, ensuring that two rows from each side remained untouched to avoid border effects. Plant growth characteristics were assessed 90 days after transplanting, utilizing five plants from each treatment/plot. The recorded characteristics included plant height, number of leaves, fresh and dry weight of the plants, number of fruits per plant, and total yield per plant, as detailed below.

• Number of leaves: After 90 days of transplanting date, the leaves of randomly selected plants were accounted and recorded.
• Plant height: It was measured from the base up to the apex of the stem of each plant in the greenhouse experiments using the gradual meter.
• Fresh and dry weight of plants: five cucumber plants were uprooted and weighed to determine their fresh weight. Then, they were placed in an air-forced drying oven set to 70 °C for 3 days to measure the dry weight of the plants using a digital balance.
• Fruit yield: Fruits were harvested once they reached a size of 10 cm in length and 3 cm in diameter. Cucumbers were harvested every two days for 120 days. The accumulated yield was expressed in gram per plant (g/plant)

### 2.6 Physiological and biochemical measurements

To evaluate physiological and biochemical responses, fully expanded leaves (the fourth leaves from the apex) were selected to ensure metabolic uniformity across treatments. To minimize within-treatment variability and enhance statistical robustness, leaf samples were collected from multiple individual plants within each replicate and pooled to form a representative composite sample for each treatment. All sampling procedures were carefully standardized to ensure consistency in the physiological status of the analyzed tissues.

#### 2.6.1 Determination of chlorophyll content

Photosynthetic pigments were determined spectrophotometrically using the method described by Arnon (1949). In brief, 0.2 g of fresh leaf samples were homogenized in 10 mL of 80% (v/v) acetone with a chilled mortar and pestle. The homogenate was then centrifuged at 5000 rpm for 10 min, and the supernatant was collected. This extraction process was repeated until the residue became colorless, after which the final volume was adjusted to a known volume with 80% acetone. The absorbance of the extract was measured with a UV-visible spectrophotometer (Shimadzu model 3700, Shimadzu Limited Company, Japan) at wavelengths of 663 and 645 nm, using 80% acetone as a blank. The concentrations of chlorophyll a, chlorophyll b, and total chlorophyll were calculated using the following equations.

• Ch1.a=12.64(A663)−2.49(A645)
• Ch1.b=5.6(A663)+23.26(A645)
• $TotalChl.=\frac{[20.2(\text{A}645+8.02(\text{A}663)]\times V}{1000\text{X}W}$

Where: A_645_= absorbance at 645nm, A_663_= absorbance at 663 nm, V= final extract volume (mL), W= fresh weight of sample (g), and 1000= conversion factor.

#### 2.6.2 Determination of hydrogen peroxide content

The technique described by Velikova et al. (2000) was utilized to evaluate the hydrogen peroxide content in cucumber leaves. In summary, 100 mg of cucumber leaves were combined with 4 mL of 0.1% trichloroacetic acid (TCA). Following centrifugation for 10 minutes at 3000 rpm, the supernatant was collected for subsequent analysis. To measure hydrogen peroxide (H_2_O_2_) in the plant leaves, 500 µL of the supernatant was mixed with 1 mL of 1 M potassium iodide (KI). The absorbance of this mixture was recorded at 390 nm using a spectrophotometer. The hydrogen peroxide content was expressed as fresh weight (nmol g^−1^ FW).

#### 2.6.3 Determination of malondialdehyde level

The MDA content in cucumber leaves was determined using the method described by Qiu et al. (2007). Fresh samples of 300 mg from each treatment were mixed with 5 mL of 5% trichloroacetic acid. The mixture was then centrifuged for 15 minutes at 8000 x g. From the obtained supernatant, 1 mL of each sample was combined with 2.5 mL of thiobarbituric acid. This mixture was heated at 100 °C in a water bath for twenty minutes and then rapidly cooled on ice. The mixture was subsequently centrifuged at 10,000 x g for 5 minutes, and the absorbance of the resulting supernatant was measured at 532 nm and 600 nm. The leaf MDA content in the cucumber plants was assessed and expressed as nmol MDA g^−1^fresh weight.

#### 2.6.4 Determination of activity level of antioxidant enzymes

A sample (0.5 g fresh tissue) was homogenized in 0.1 M sodium phosphate buffer (pH 7.1) at a ratio of 2 mL buffer per gram tissue using a chilled mortar and pestle. The homogenate was filtered through four layers of cheesecloth and centrifuged at 12,000 rpm for 20 min at 5 °C. The resulting supernatant was used as the crude enzyme extract for determination of polyphenol oxidase (PPO) and peroxidase (POD) activities using a spectrophotometer. PPO activity was determined according to Matta and Dimond (1963) using catechol as substrate. The reaction mixture contained enzyme extract, sodium phosphate buffer (0.2 M, pH 7.0), and 1 mM catechol, and the increase in absorbance was recorded at 495 nm every 30 s for 2 min. PPO activity was calculated as the rate of change in absorbance per minute (ΔA495 min^−1^). POD activity was assayed according to Allam and Hollis (1972) by measuring the oxidation of pyrogallol in the presence of H_2_O_2_. The reaction mixture contained sodium phosphate buffer (pH 7.0), pyrogallol, H_2_O_2_, and enzyme extract, and the increase in absorbance was recorded at 425 nm. POD activity was calculated as the rate of change in absorbance per minute (ΔA425 min^−1^). Thus, both PPO and POD activities were determined using kinetic (rate-based) spectrophotometric assays rather than endpoint measurements. Enzyme activities were expressed as specific activities normalized to protein content (U mg^−1^ protein), where protein concentration was determined using the Bradford method (Bradford, 1976), to ensure accuracy and comparability among treatments.

#### 2.6.5 Determination of total free amino acids

Total free amino acids (TFFA) were estimated using methods reported by Hu et al. (2021). Briefly, 5 g of fresh leaf samples were homogenized in 5 mL of 80% ethanol. The homogenate was then centrifuged at 10,000 x g for five minutes. The supernatant was used to measure the free amino acid content. Free amino acids were measured using an automatic amino acid analyzer (Hitachi L-8900, Hitachi High-Technologies, Japan).

#### 2.6.6 Determination of proline content

The leaf proline content in cucumber plants was assessed according to the technique stated by Dobra et al. (2011) with slight modifications. Briefly, 200 mg fresh weight of cucumber leaves was taken from each treatment and homogenized in 10 ml of aqueous sulfosalicylic acid. The homogenate was filtered using Whatman filter paper (No. 1). One millimeter of filtrate was combined with an aqueous solution that included one milliliter of acid-ninhydrin reagent and one milliliter of glacial acetic acid in a test tube. Thereafter, the mixture-filled test tube was placed in the water bath and heated for 60 minutes at 100 °C. The test tube was inserted in an ice bath to halt the reaction. Five millimeters of toluene were added in the test tube, then shaken by hand, and finally incubated at room temperature for 15 min. The absorbance of the upper layer of the mixture containing toluene was measured at 520 nm, with toluene as a blank control.

#### 2.6.7 GC-M Sanalysis of applied essential oils

Samples of garlic and cumin oils were diluted in n-hexane to a final concentration of 1% (v/v), vortex-mixed, and filtered through a 0.22 µm PTFE membrane filter prior to injection. A 1 µL aliquot was injected in split mode. GC–MS analysis was performed using an Agilent 6890N GC-MScoupled with an Agilent 5973N mass selective detector (MSD), equipped with a DB-5MS capillary column (30 m × 0.25 mm i.d., 0.25 µm film thickness; J&W Scientific, Folsom, CA, USA). The oven temperature was programmed with a stepwise heating ramp to ensure efficient separation of both low- and high-boiling volatile compounds. The temperature was initially held at a low value, then increased at controlled ramp rates to the final temperature, with holding periods at intermediate and final stages to enhance peak resolution and minimize co-elution. Helium was used as the carrier gas. Two flow rates (1.0 and 1.5 mL min^−1^) are mentioned because preliminary optimization runs were conducted at 1.0 mL min^−1^, whereas confirmatory analyzes were performed at 1.5 mL min^−1^. The slightly higher flow rate improved peak symmetry and signal intensity for later-eluting compounds without compromising chromatographic resolution. The MS was operated in electron impact (EI) mode at 70 eV, with an ion source temperature of 230 °C and a transfer line temperature of 280 °C. The ionizing current was 200 mA, the detector gain was set at 800 V (maximum 1.5 kV), and mass spectra were recorded over a scan range of 25–800 amu at a scan speed of 0.45 s per scan. Compound identification was achieved by comparison of mass spectra with those in the NIST mass spectral library and with authentic reference standards where available. Only identifications with a similarity index (SI ≥ 90%) were accepted. Retention indices (RI) were also calculated and compared with literature data to support compound assignment. Relative component percentages were determined by peak area normalization and expressed as percentages of the total chromatographic area.

### 2.7 Molecular analyzes and computational analysis

#### 2.7.1 Differential gene expression analyzes using quantitative real-time polymerase chain reaction

Two stress-responsive genes were identified: Pathogenesis-related gene 1 (*PR-1*) and linoleate 9S-lipoxygenase 6-like (*LOX-1*). Transcript analysis of these genes was performed using quantitative real-time PCR. These genes have been shown to respond to the powdery mildew pathogen in cucumber plants. RNA was extracted from treated plants exposed to garlic and cumin oil after 24 hours of infection process of powdery mildew were investigated. Samples were immediately frozen in liquid nitrogen after harvesting and stored at −80 °C until further use. Total RNA was extracted from 100 mg of cucumber plant tissue using the RNeasy^®^ Plant Mini Kit (Qiagen, Hilden, Germany), following the manufacturer’s instructions. A no-template control (NTC) was included in all RT-qPCR assays to detect potential contamination and non-specific amplification; no amplification was observed in these controls, confirming the specificity of the reactions. RNA purity was assessed using spectrophotometric measurements, and only samples with acceptable A260/A280 and A260/A230 ratios were included. In addition, RNA integrity was verified by agarose gel electrophoresis, where intact ribosomal RNA bands confirmed the absence of degradation. Between 1 and 2 μg of DNase I-treated RNA was reverse transcribed using the Prime-Script First Strand cDNA Synthesis Kit (Thermo Kit). cDNA was analyzed via quantitative real-time PCR using the SYBR kit (Takara) in the Thermal Cycler Bio-Rad Real-Time System II (Bio-Rad, California, USA). Primers specific to PR-1 and LOX-1 were designed using Primer Blast (https://www.ncbi.nlm.nih.gov/tools/primer-blast/) and Primer3 (https://primer3.ut.ee/) software, as shown in **Table 1**. The primers were employed in RT-qPCR to detect fragments approximately 80–200 bp in length, using the Takara SYBR^®^ Premix Ex Taq™ in 25 μl reactions that contained 60 ng of template cDNA, 5 μl of each 1 μmol/l oligonucleotide, and 12.5 μl SYBR premix ExTaq. Amplification involved an initial denaturation at 95 °C for 120 seconds, followed by 40 cycles of 95 °C for 15 seconds and 60 °C for 30 seconds. Melting curves were generated for each data point to verify the specificity of the PCR products. To analyze gene expression results, the delta-delta Ct method was employed. Average CT values were determined from triplicate experiments for each gene, calculated by subtracting the average CT value of the genes from the CT value of the Alaska alpha-tubulin gene. Tubulin served as a housekeeping gene to normalize cDNA concentrations.

**Table 1:** Primers used for quantitative real time -PCR analysis of reference and targeted genes.

Abbreviation	Gene	Forward primer (5–3)	Reverse primer (3–5)	Accession no.
PR-1	pathogenesis-related protein 1	TTGTGGGTGGATG-AGAAGCC	AATGACCACACAAC-TCGCCA	XM_011660558.2
LOX-1	linoleate 9S-lipoxygenase 6-like	CTCTTGGGTGGT-GGTGTTTC	TGGTGGGATTGAAGT-TAGCC	>NM_001305730.1
Actin	House Keeping	TGCTGGTCGTG-ACCTTACTG	GAATCTCTCAGCTCC-GATGG	XM_011659465.2

#### 2.7.2 Molecular docking analysis

Molecular docking of the biological treatment compounds tested was conducted using AutoDockVina software (42) to analyze the interactions between the target virulence proteins of *P. xanthii* and the ligand structures of the biological treatment compounds. This analysis aimed to determine the direct effects of these compounds on the inhibition of *P. xanthii*. The Chitin synthase (CHS) sequence (accession: A0A068FR90) was retrieved from the National Center for Biotechnology Information (NCBI) protein database. The direct link to this protein entry is https://www.uniprot.org/uniprotkb/A0A068FR90/entry for building binding models with a three-dimensional(3D) structure, utilizing the AlphaFold Protein Structure Prediction Server. This server employs a high-end deep-learning algorithm designed to predict high-confidence 3D models from FASTA sequences. The target protein sequence from Pseudoperonospora cubensis, the affinity minimization of the targeted protein was performed using the 3DREFINE server (http://sysbio.rnet.missouri.edu/3Drefine/index.html). To obtain targeted ligands of essential oils for molecular docking, GC-Mass analysis databases were utilized. Ligands were downloaded in SDF format from sites like PubChem (https://pubchem.ncbi.nlm.nih.gov/), and Open Babel software (http://openbabel.org/wiki/Main_Page) was used to change them into MOL2 format. All 3D structures of the ligands were energy-minimized using Avogadro 1.2.0 software (43), applying the MMFF94 force field to generate optimized conformations suitable for molecular docking experiments. Prior to docking, all water molecules and ligands were removed, and hydrogen atoms were added to the target proteins. We analyzed the interaction of the constructed protein model (CasA3) with the ligand structures of the biological treatment compounds. Docking of the targeted protein against the studied compounds was facilitated by AutoDockVina software (42). Binding free energy calculations were carried out using the scoring function of AutoDockVina as part of its script. After an exhaustive search, 100 poses were evaluated, and the best-scoring poses were selected to calculate the binding affinity of the ligands. Additionally, Discovery Studio (https://www.uniprot.org/uniprotkb/A0A068FR90/entry) was employed to visualize the 2D structures of the ligands.

### 2.8 Statistical analysis

The data were analyzed using Statistica 7 software (model 2007). A combined analysis was performed across seasons after assessing the normal distribution of errors with a Shapiro–Wilk test and evaluating homogeneity of variance using Levene’s test. A two-way analysis of variance (ANOVA) was conducted to determine the significance of variation. Means were compared using the Duncan range test at a significance level of p< 0.05. The means in tables and figures shown the interaction between the treatments and seasons. Additionally, the figures were created using Google Colab (https://colab.research.google.com/#scrollTo=C4HZx7Gndbrh), while the heatmap correlations were generated using the online tool SRplots (https://www.bioinformatics.com.cn/en).

## 3 Results

### 3.1 Chemical characterization of violate compounds of used essential oils

GC-MSanalysis of garlic (*Allium sativum* L.) and cumin (*Cuminumcyminum* L.) essential oils revealed a complex mixture of bioactive organic compounds, including alcohols, aldehydes, fatty acids, hydrocarbons, esters, and heterocyclic compounds. In total, 54 compounds were identified in garlic oil and 62 in cumin oil. Some of these constituents in garlic oil i.e. Cyclohexene,4-(4-ethylcyclohexyl)-1-penty, Methyldiethylborane and Heptadecane (**Supplementary Table 1**). While, some of these constituents in in cumin oil i.e. Cyclohexene,1-methyl-4- (1-methylethylidene),b,10- tetrahydro-8-methoxy -5-methylindeno[1,2-b]indol,9,10-9,10-Anthracenedione, 1,4-diamino-2- methoxy-,Trans-caryophyllene bicyclo[3.1.1] hepta -2-ene,2,6 -dimethyl-6- (4-methyl-3-pentenyl) and Aromadendrene (**Supplementary Table 2**).

### 3.2 Disease severity

**Figure 1:**
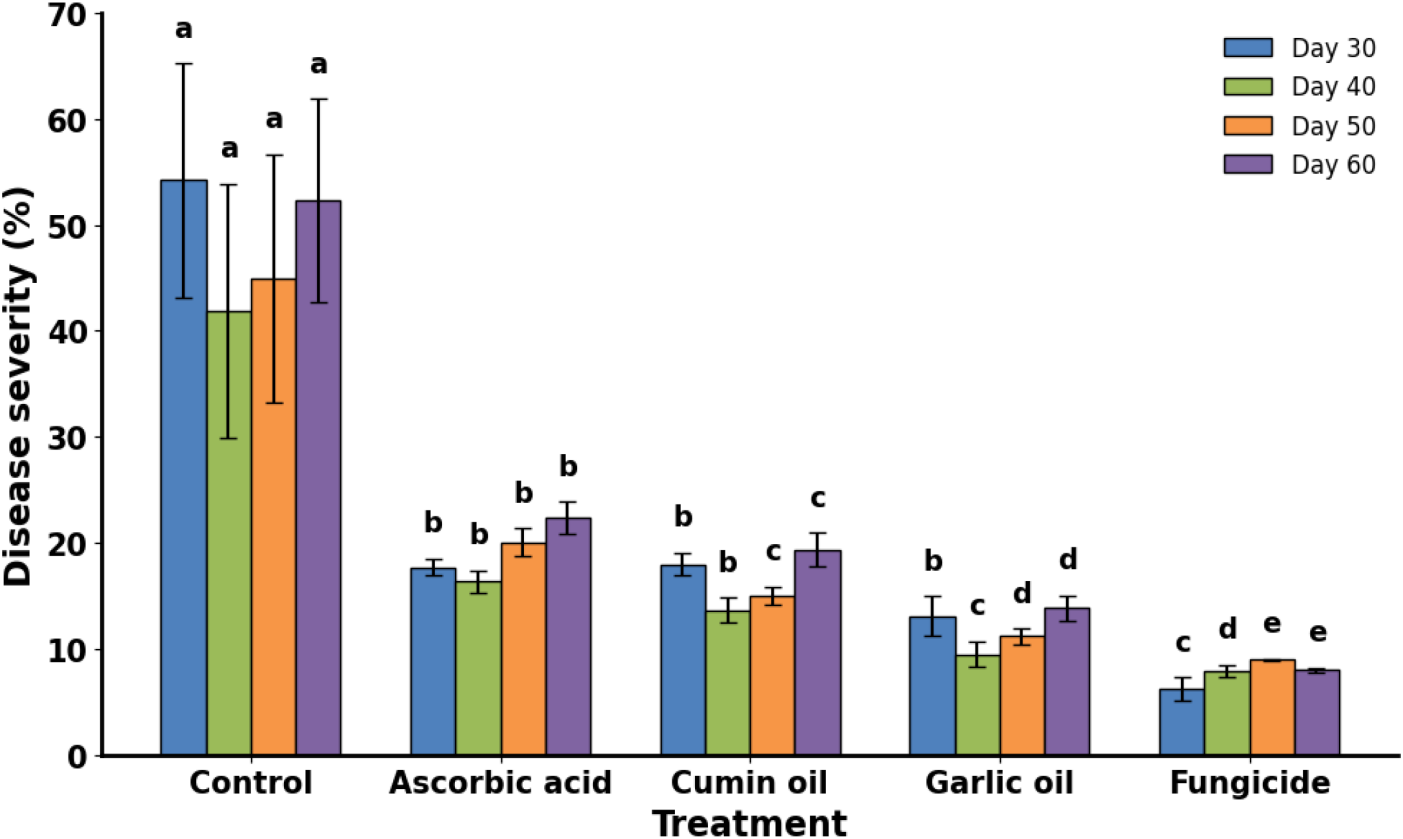
Effect of essential oils, ascorbic acid and chemical fungicide on the disease severity of powdery mildew on the cucumber plant. Columns followed by different letter(s) are significantly different at p ≤ 0.05 according to Duncan test. The vertical bar represents the standard error (n = 10).

The data in **Figure 1** illustrates the impact of essential oils and the fungicide ascorbic acid on the severity of powdery mildew disease in cucumber plants at 30, 40, 50, and 60 days post-treatment. Significant differences were noted among the treatment means. A notable decline in disease severity was observed in all treated plants compared to the untreated control. The untreated plants exhibited the highest disease severity values, which remained significantly higher than those recorded in the treated plants (p ≤ 0.05) throughout the experimental period. In contrast, the fungicide application demonstrated the most pronounced suppression of disease severity, followed by garlic oil, cumin oil, and ascorbic acid when compared to the control over time. Furthermore, the application of cumin oil, garlic oil, ascorbic acid, and fungicide treatments significantly reduced the severity of powdery mildew by 79.18%, 85.93%, 87.05%, and 87.05%, respectively. Overall, all treatments significantly reduced powdery mildew severity compared with the untreated control across all assessment dates, with the chemical fungicide and garlic oil being the most effective, followed by cumin oil and ascorbic acid.

### 3.3 Progress of powdery mildew disease

**Figure 2:**
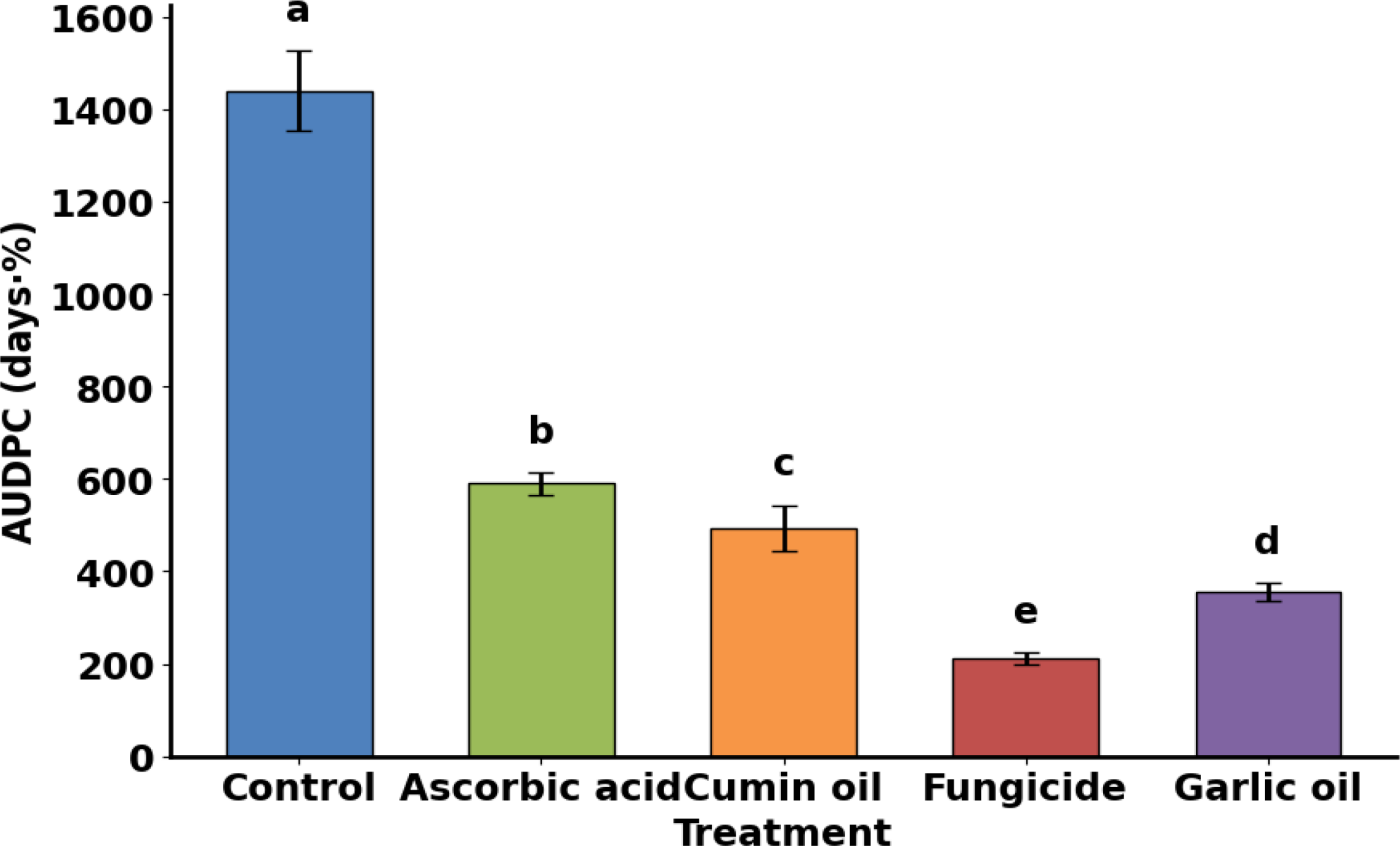
Effect of essential oils, ascorbic acid and chemical fungicide on area under the disease progress curve (AUDPC) for powdery mildew in cucumber plants. Columns followed by different letter(s) are significantly different at p ≤ 0.05 according to Duncan test. The vertical bar represents the standard error (n = 10).

**Figure 2** clarifies the influence of essential oils and the fungicide ascorbic acid on the area under the disease progress curve (AUDPC) for powdery mildew in cucumber plants. The untreated plants, which served as the control treatment, exhibited the highest area under the disease progress curve (AUDPC) value, indicating a rapid and severe spread of the disease during the experiment. In contrast, all treatments significantly reduced the AUDPC values compared to the untreated plants (p ≤ 0.05). The fungicide treatment yielded the lowest AUDPC value, followed by garlic oil, cumin oil, and ascorbic acid in that order. When compared to the untreated plants, the AUDPC showed reductions of -62.33%, -71.21%, -60.61%, and -84.88% with cumin oil, garlic oil, ascorbic acid, and fungicide, respectively. This result is consistent with the findings on disease severity, emphasizing the enhanced protective effectiveness of both the fungicide and the essential oils.

### 3.4 Morphological parameters

**Table 2** presents the effectiveness of essential oils, ascorbic acid, and chemical fungicide on the growth parameters and chlorophyll content of cucumber plants infected with powdery mildew. The results demonstrated that all the treatments applied substantially improved the plant growth than the control treatment. The control plants displayed the lowest measurements in terms of plant height, number of leaves, fresh weight, dry weight and leaf chlorophyll content (a, b, and total chlorophyll). This observation underscores the detrimental effect of powdery mildew on the morphological characteristics of the untreated plants. Conversely, the greater values for pervious growth parameters were recorded in the plants treated with garlic oil and chemical fungicide treatments, with no statistically significant differences among them. The utilization of cumin oil and ascorbic acid also increased the growth parameters relative to control treatment, but lower than the garlic oil treatment. Regarding the leaf chlorophyll content, the treatment of cumin oil, garlic oil, ascorbic acid and chemical fungicide significantly increased the concentration of leaf Chl a, Chl b, and total Chl. compared with the untreated plants, without any significant changes among the treatments.

**Table 2:** Effect of essential oils, ascorbic acid and chemical fungicide on vegetative growth and chlorophyll content.

Treatment	Plant height	Plant FW	Plant DW	No. leaves	Chl.*a*	Chl.*b*	Total Chl.
	(cm)	(g)	(g)	-	(mg.g^-1^ leaves FW)		
**Control**	1.42±0.21 **d**	190.50±6.83**e**	34.5±3.8 **d**	24.5±3.44**c**	2.49±0.20 **b**	1.40±0.13**b**	3.89±0.32**b**
**Ascorbic acid**	2.01±0.057**c**	290.67±7.42 **d**	67±4.04 **c**	36.5±5.01**b**	4.54±0.38**a**	2.29±0.16**a**	6.83±0.45**a**
**Cumin oil**	2.12±0.098**b**	297.50±6.89 **c**	69±3.58 **b**	39±3.57**ab**	4.26±0.18**a**	2.52±0.47**a**	6.78±0.65**a**
**Fungicide**	2.20±0.071**a**	318.1±9.52**a**	74.0±3.6 **a**	42±2.99**a**	4.91±0.06**a**	2.36±0.03**a**	7.26±0.08**a**
**Garlic oil**	2.33±0.126**a**	308.83±6.30 **ab**	72.5±3.8 **a**	43.5±3.50**a**	4.41±0.23 **a**	2.63±0.28**a**	7.04±0.51**a**

FW, fresh weight, DW, dry weight.

Means followed by different letter(s) are significantly different at p ≤ 0.05 according to Duncan test.± values represents the standard error (n = 10).

### 3.5 Fruit yield and its components

**Figure 3:**
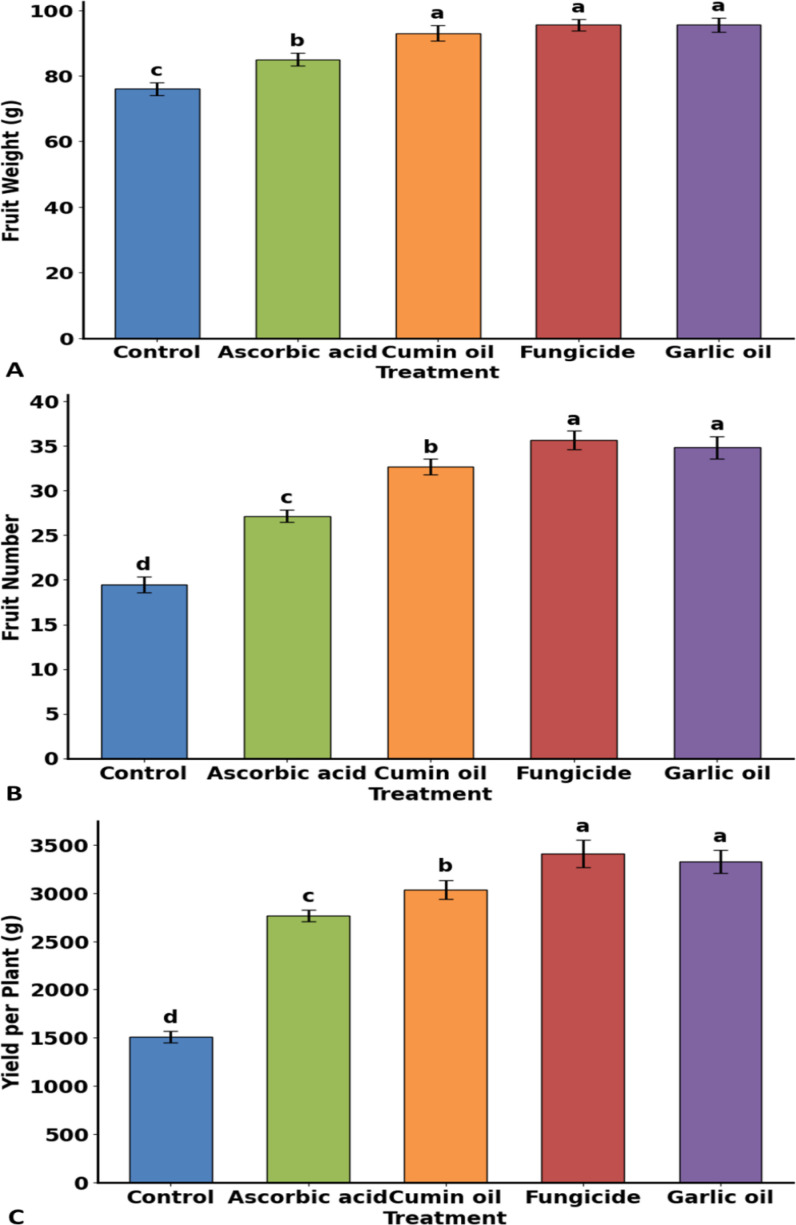
Effect of essential oils, ascorbic acid and chemical fungicide on fruit weight **(A)**, fruit number **(B)** and total yield **(C)** of cucumber plants infested with powdery mildew disease. Columns followed by different letter(s) are significantly different at p ≤ 0.05 according to Duncan test. The vertical bar represents the standard error (n = 6).

**Figure 3** shows the changes in the fruit weight, fruit number, and total yield between treatments applied to cucumber plants infected with powdery mildew. The maximum fruit weight was observed in cucumber plants treaded with garlic oil, cumin oil, and chemical fungicides treatments followed by ascorbic acid and the lowest fruit weigh recorded in the untreated plants. A similar pattern emerged concerning fruit quantity; specifically, the applications of garlic oil and chemical fungicide produced the highest number of fruits compared to the other treatments (**Figure 3B**). Cucumber plants treated with cumin oil and ascorbic acid showed a moderate improvement, while the untreated specimens had the lowest fruit production. As a result, all treatments significantly increased the total fruit yield when compared to the untreated control (**Figure 3C**). The highest fruit yield was recorded in cucumber plants treated with chemical fungicide (3408.17 g) and garlic oil (3327.33 g). Following these, cumin oil (3037 g) and ascorbic acid (2866.5 g) were applied. In contrast, the untreated plants yielded the least, at 1509 g.

### 3.6 Hydrogen peroxide content and malondialdehyde

**Figure 4:**
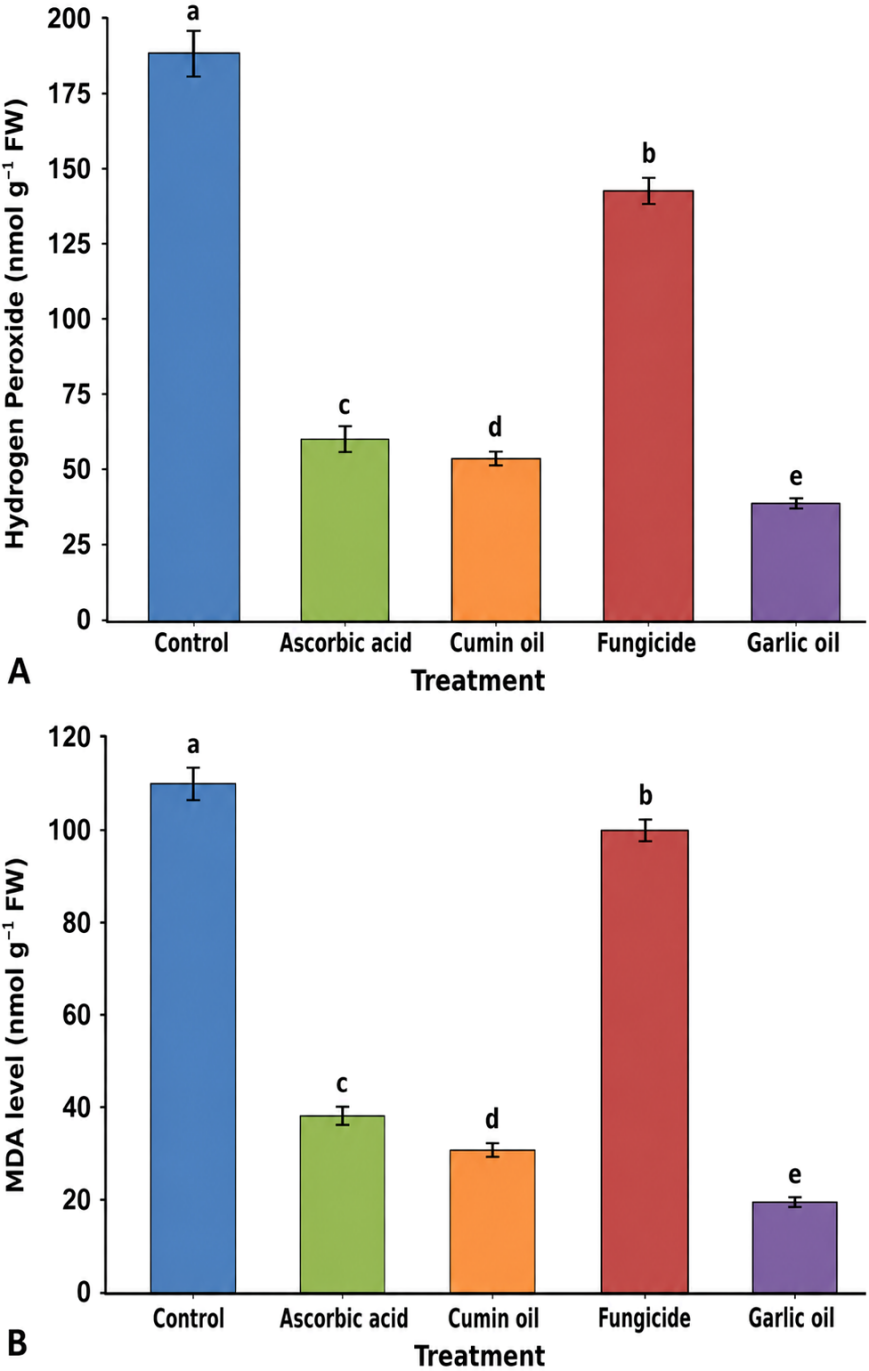
Effects of different treatments on oxidative stress markers in plants. Hydrogen peroxide [H_2_O_2_, **(A)**] content (nmol g⁻¹ FW). Malondialdehyde [MDA, **(B)**] level (nmol g⁻¹ FW). Values represent mean ± SE. Different letters above bars indicate significant differences among treatments according to Tukey's test at P ≤ 0.05. The vertical bar represents the standard error (n = 6).

The data indicate that the treatments applied significantly influenced the levels of H_2_O_2_ and MDA, as shown in **Figures 4A, B**. The maximum activity levels of H_2_O_2_ (180 mmol g^−1^ FW) and MDA (115 nmol g^−1^ FW) were found in the untreated plants compared to all other treatments. In contrast, all the treated cucumber plants exhibited significantly lower levels. The lowest levels of H_2_O_2_ and MDA were observed in the plants treated with garlic oil, followed by those treated with cumin oil, ascorbic acid, and chemical fungicide. The reduction ratios of H_2_O_2_ in the treated plants were -69.36%, -72.61%, -24.26%, and -77.23%, while the reductions in MDA levels were -69.0%, -71.31%, -8.42%, and -77.24% for the plants treated with ascorbic acid, cumin oil, chemical fungicide, and garlic oil, respectively.

### 3.7 Antioxidant enzymes

**Figure 5:**
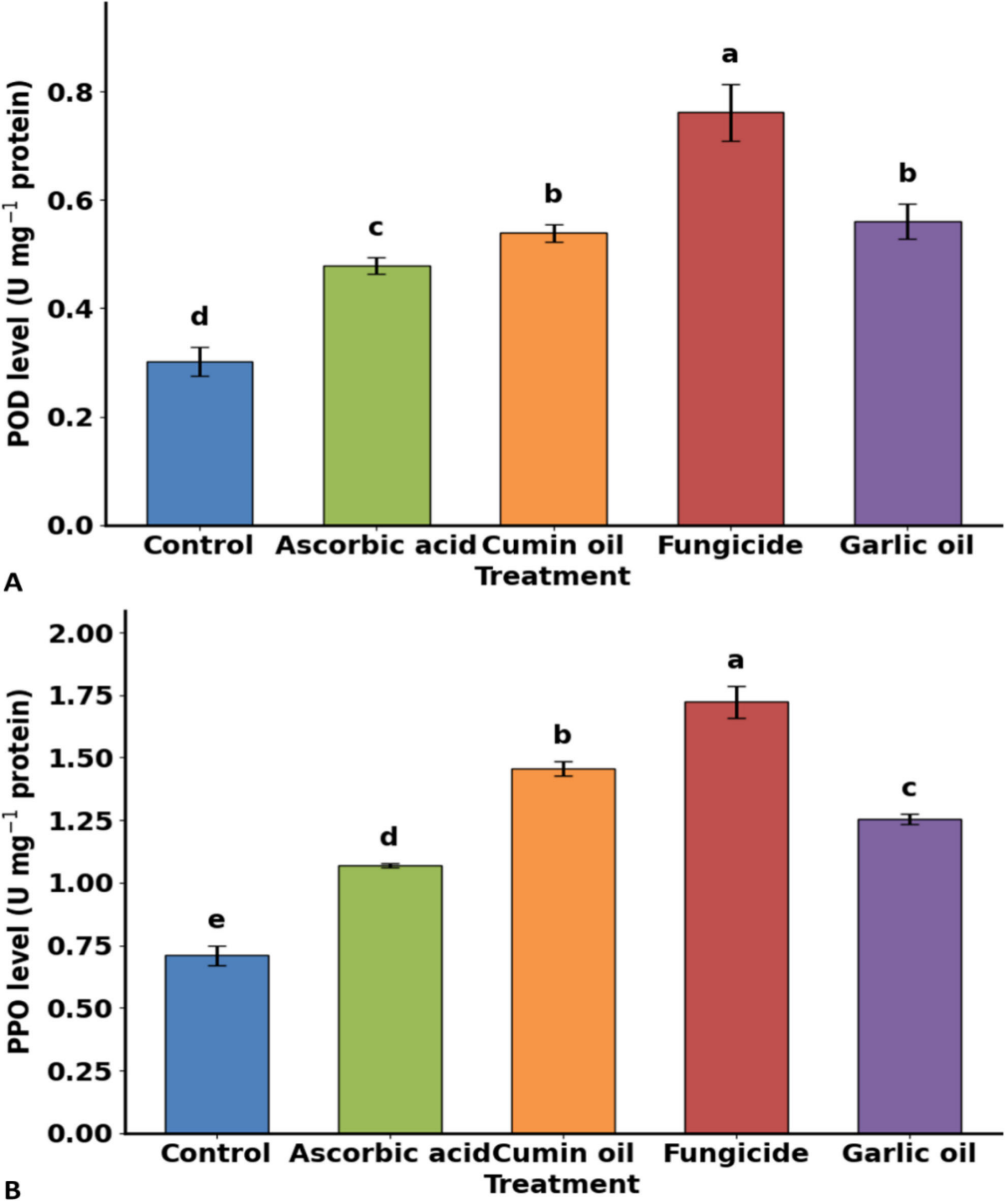
Effect of essential oils, ascorbic acid and chemical fungicide on peroxidase [POD, **(A)**], and polyphenol oxidase [PPO, **(B)**] of cucumber leaves infested with powdery mildew disease. Columns followed by different letter(s) are significantly different at p ≤ 0.05 according to Duncan test. The vertical bar represents the standard error (n = 6).

The treatment applied positively influenced the activities of antioxidant enzymes, specifically POD and PPO, as shown in **Figures 5A, B**. The highest levels of POD and PPO activities were found in the plants treated with chemical fungicide, followed by those treated with garlic oil, cumin oil, and ascorbic acid. In contrast, control plants showed the lowest activity levels of these antioxidant enzymes (POD and PPO). The maximum enzyme activity for POD and PPO was observed in cucumber plants treated with chemical fungicide, followed by those treated with cumin oil, garlic oil, and ascorbic acid. The minimum activity levels of POD and PPO were recorded in the control plants.

### 3.8 Biochemical compounds

**Figure 6:**
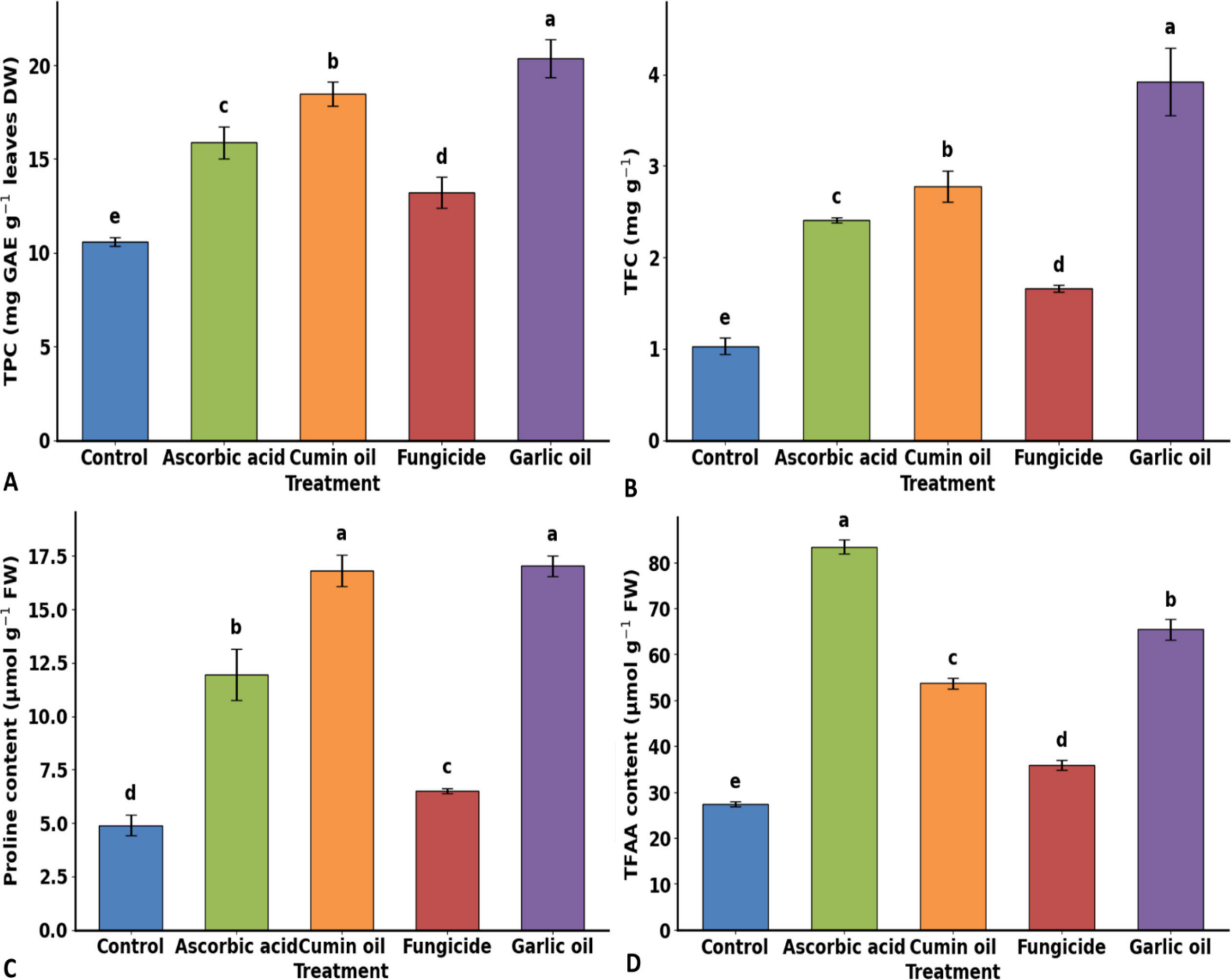
Effect of essential oils, ascorbic acid and chemical fungicide on total phenol content [**(A)**, PPO], total flavonoids content [**(B)**, TFC], proline content **(C)**, and total free amino acids [TFAA, **(D)**]. Columns followed by different letter(s) are significantly different at p ≤ 0.05 according to Duncan test. The vertical bar represents the standard error (n = 6).

The biochemical compounds in cucumber leaves infected with powdery mildew demonstrated positive enhancements following the foliar application of essential oils, ascorbic acid, and fungicide, as shown in **Figures 6A–D**. All treatments significantly increased the total TPC, TFC, proline, and tTFAA in treated plants compared to the untreated control treatment. Garlic oil yielded the highest levels of TPC (21 mg GAE/g DW), total flavonoids (4.2 mg/g FW), proline (20 µmol/g FW), and TFAA (65 µmol/g FW), followed by cumin oil, ascorbic acid, and fungicide treatments. On the contrary, the control treatment exhibited the lowest values. The garlic oil treatment resulted in increases in TPC, TFC, proline, and TFAA by 43.08%, 67.22%, 65.33%, and 53.55%, respectively. The statistical analysis confirmed that natural essential oils, particularly garlic and cumin oils effectively enhanced the antioxidant and osmoprotectant responses in cucumber plants infested with powdery mildew.

### 3.9 Relative gene expressions of linoleate 9S-lipoxygenase 6-like and pathogenesis-related 1

**Figure 7:**
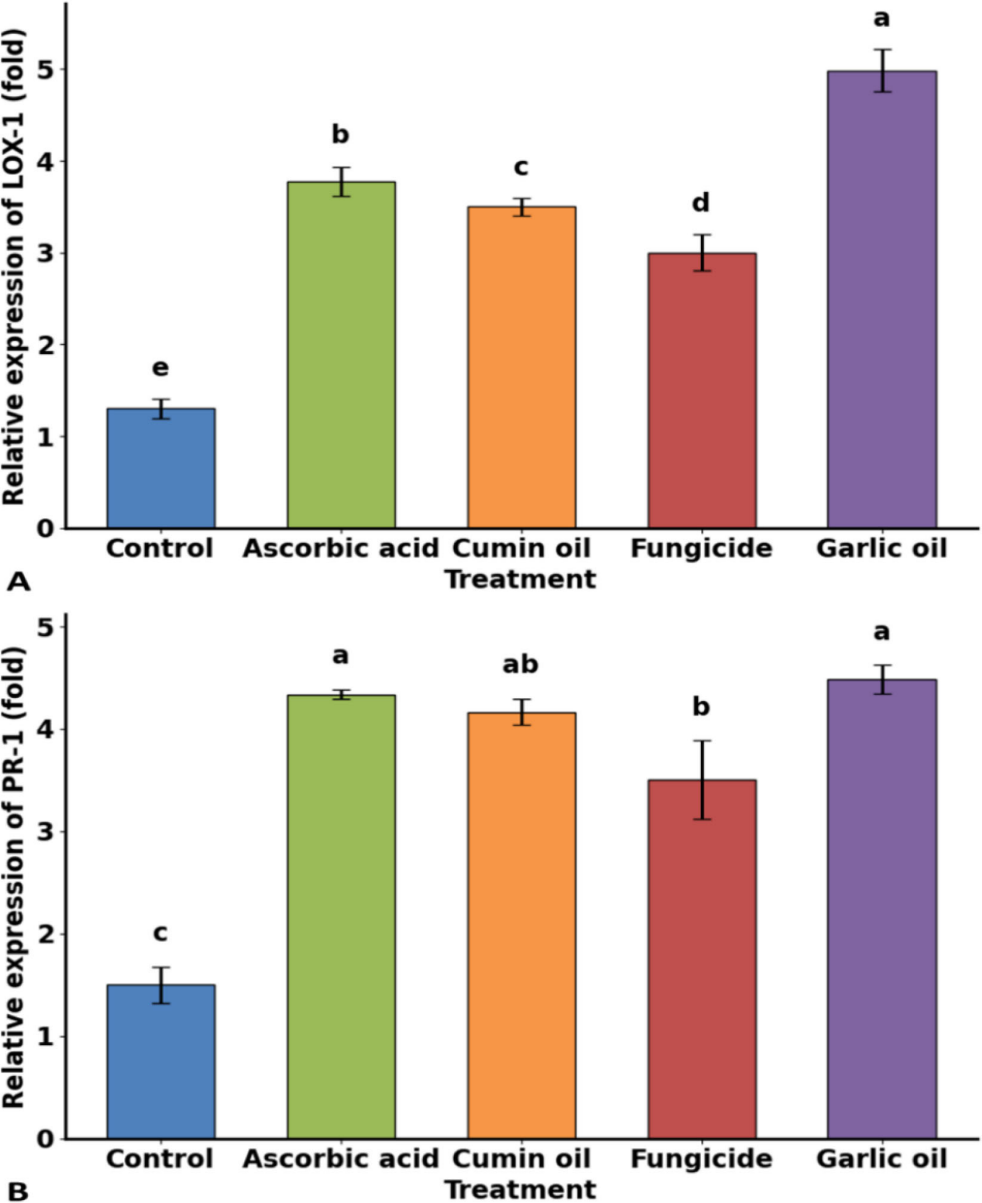
Effect of essential oils, ascorbic acid and chemical fungicide on relative gene expressions of LOX-1 **(A)** and PR-1 **(B)**. Columns followed by different letter(s) are significantly different at p ≤ 0.05 according to Duncan test. The vertical bar represents the standard error (n = 6).

The result of gene expression analysis in **Figures 7A, B** revealed the significant differences among treatments (p ≤ 0.05). Garlic oil and cumin oil treatments strongly induced the expression of both genes under powdery mildew infection, whereas control plants (untreated plants infected with the pathogen) exhibited the lowest expression levels. Specifically, LOX-1 expression increased 5.0-fold in garlic oil-treated plants and 3.5-fold in cumin oil-treated plants relative to the untreated control infected with the pathogen. Similarly, PR-1 expression was highest in garlic oil-treated plants (4.5-fold), followed by cumin oil-treated plants (4.0-fold). Ascorbic acid and chemical fungicide also enhanced gene expression but to a lesser extent. Garlic oil treatment showed the strongest induction of LOX-1 and PR-1, increasing expression by 73.9% and 66.5%, respectively, compared to the control. These differential expression patterns indicate that garlic and cumin oils effectively stimulate cucumber defense pathways, likely through the activation of salicylic acid–mediated (LOX-1) and (PR-1) signaling.

### 3.10 Effects of garlic and cumin essential oil compound on the virulence proteins of *P. xanthii* using molecular docking analysis

**Figure 8:**
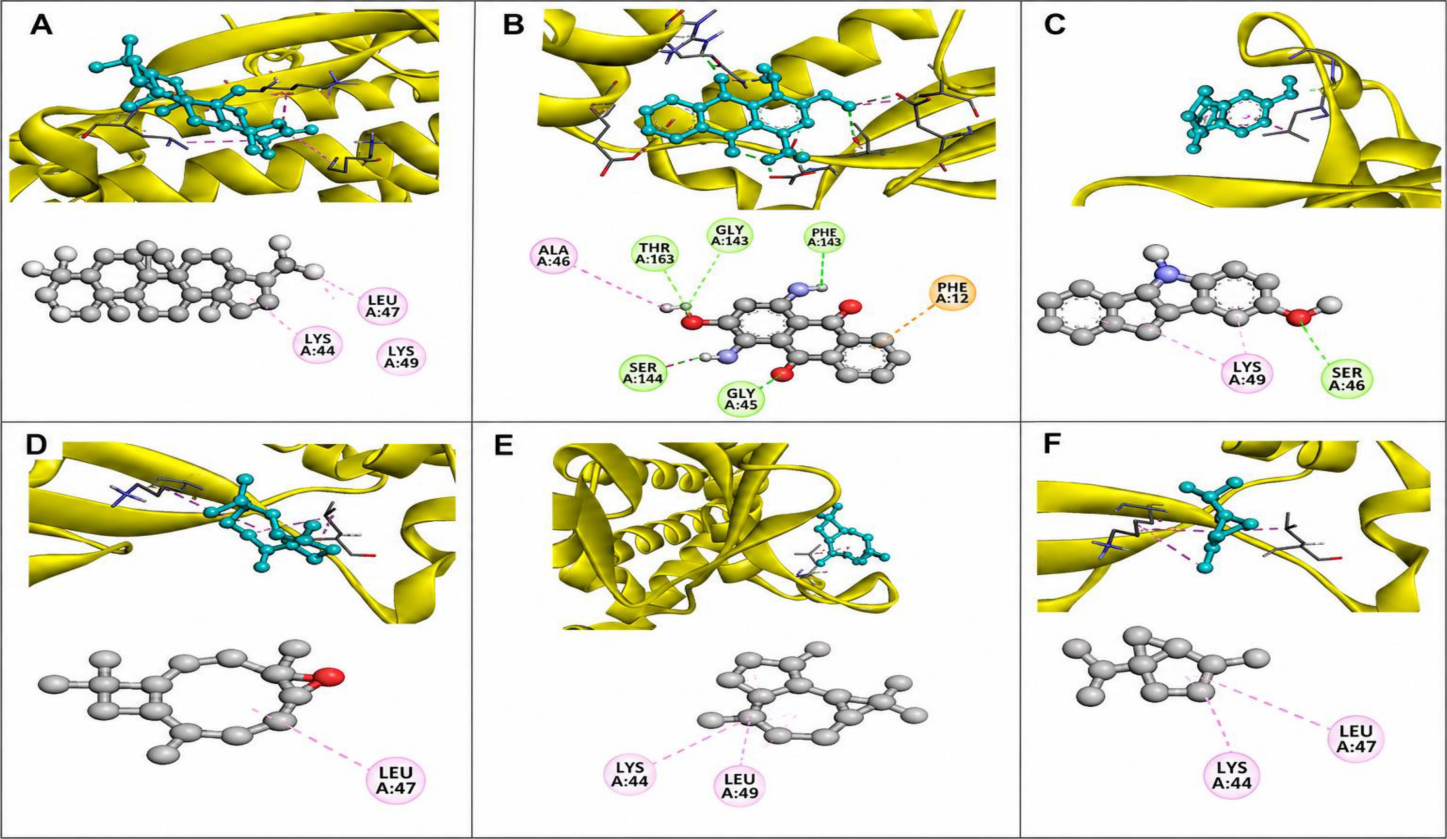
The 2D and 3D molecular interaction diagrams showing the binding modes of the virulence protein chitin synthase with major bioactive compounds identified in cumin oil. **(A)** Cyclohexene, 1-methyl-4-(1-methylethylidene), forming interactions with Lysine, **(B)** 9,10-anthracenedione1,4-diamino-2-methoxy interacting primarily with alanine residues; and **(C)** 9b,10-tetrahydro-8-methoxy-5-methylindeno[1,2-b]indol interacting with glycine, **(D)** Aromadendrene interacting primarily with leucine, **(E)** Caryophyllene oxide interactions with lysine and **(F)** Bicyclo[3,1,0]hepta-2-enomythyl-5-(1-methylethyl) associated residues within the active site pocket.

**Figure 9:**
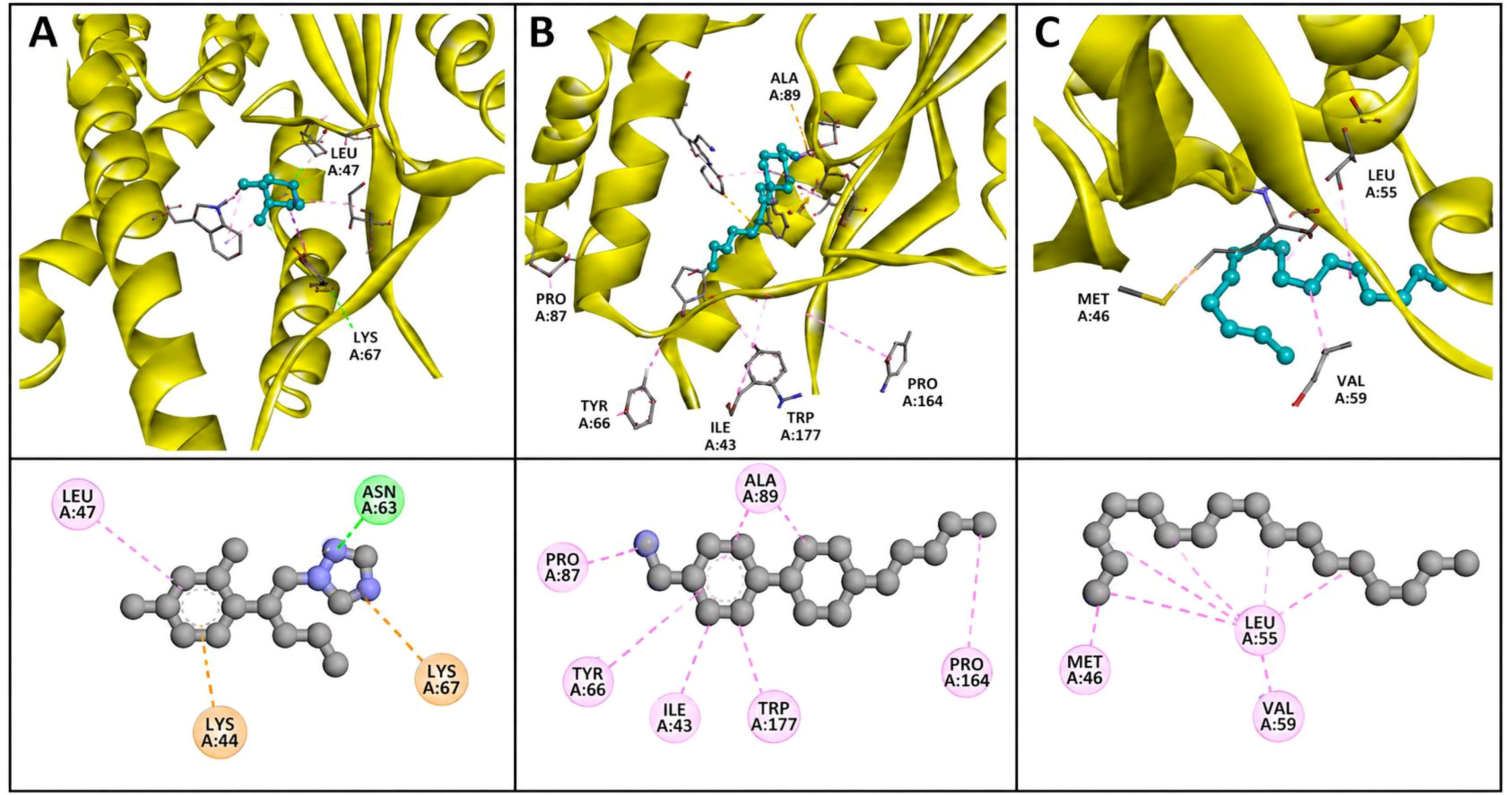
The 2D and 3D molecular interaction diagrams illustrating the binding modes of the virulence protein chitin synthase with major bioactive compounds identified in garlic oil. **(A)** Cyclohexene,4-(4-ethylcyclohexyl)-1-pentyl, pentyl, interacting with lysine **(B)** 1-Methyl-2-methylenecyclohexane, interacting with alanine and **(C)** Heptadecane interacting with leucine residues and related residues within the active site pocket.

**Figure 10:**
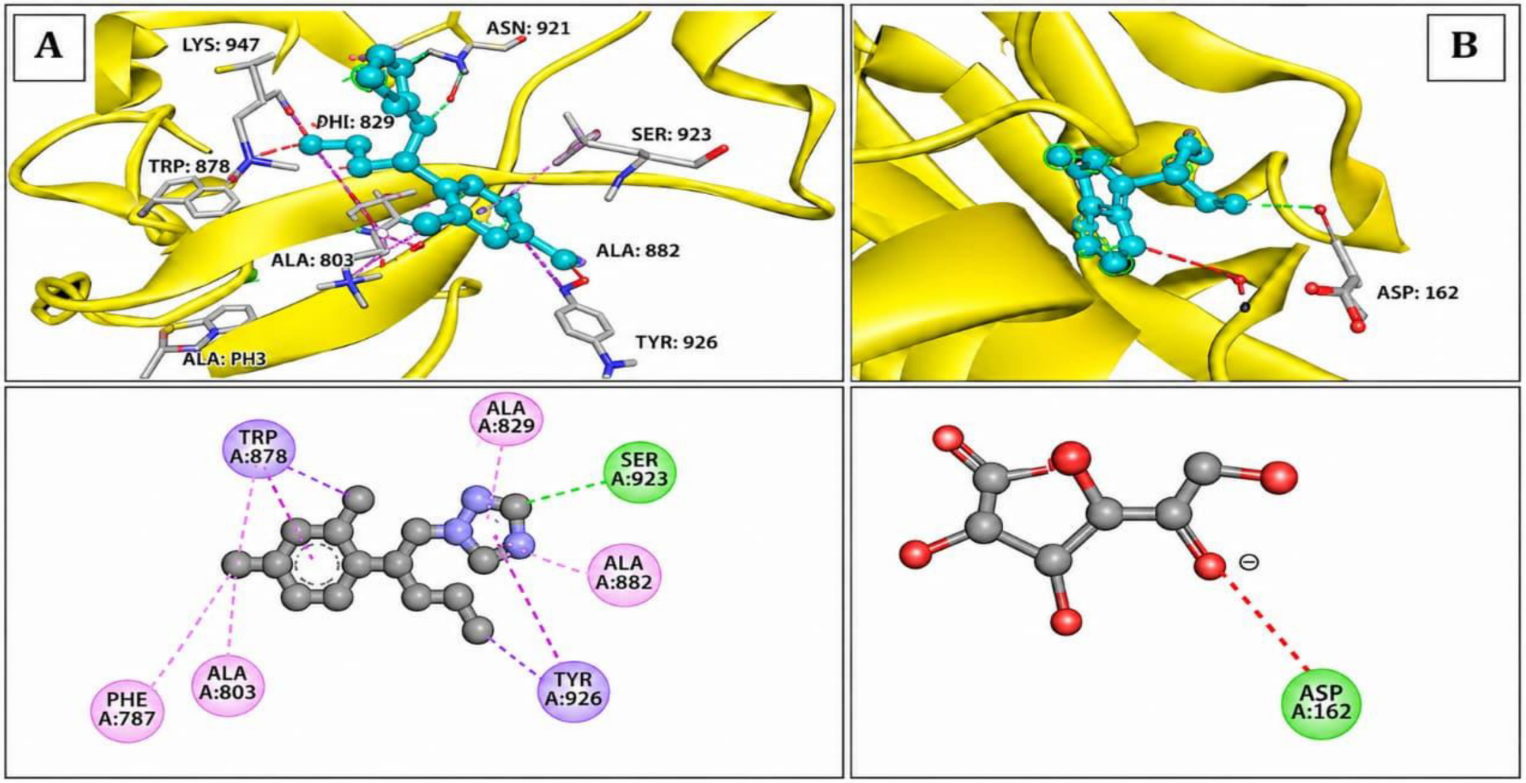
The 2D and 3D molecular interaction diagrams showing the binding mode of the virulence protein chitin synthase with **(A)** fungicide (Topas^®^) interactions with alanine, ascorbic acid **(B)** interactions with aspartic acid (ASP162) residues within the active site pocket of the protein.

The *P. xanthii* proteins that were planned in relation to the active compounds present in cumin and garlic essential oils were docked (**Tables 3**, **4**; **Figures 8**, **9**). This investigation was conducted to determine the ligands expected to block their activities and, hence, to gain a better explanation of their mode of action in controlling the pathogenicity of the *P. xanthii* isolate. Key virulence-associated proteins, notably chitin synthase, were modeled. This vital protein plays a crucial role in fungal cell wall biosynthesis, thereby representing a promising target for antifungal compounds (**Figures 8**, **9**).”The data presented in **Table 3** and **Figure 8** indicate that Cyclohexene, 1-methyl-4-(1-methylethylidene), 9,10-Anthracenedione1,4-diamino-2-methoxy, 9b,10-tetrahydro-8-methoxy-5-methylindeno[1,2-b]indol, Aromadendrene, Caryophyllene oxide and Bicyclo[3,1,0]hepta-2-enomythyl-5-(1-methylethyl) from cumin oil exhibited binding affinities with the Chitin_synthase protein, ranging from -8.4 to -5.8 kcal/mol. Furthermore, the information in **Table 4** shows that Cyclohexene,4-(4-ethylcyclohexyl)-1-pentyl-1-Methyl-2-methylenecyclohexane and Heptadecane from garlic essential oil achieved the highest docking scores with the Chitin_synthase protein, with binding energies between -7.5 and -5.8 kcal/mol (**Figure 9**). In contrast, ascorbic acid and fungicide (Topas^®^) demonstrated docking scores with the Chitin_synthase protein, yielding a binding energy of -7.9 and -6.6 kcal/mol, respectively (**Table 4** and **Figure 10**). This finding suggests that these compounds may inhibit fungal growth by targeting Chitin synthase, a crucial enzyme involved in cell wall formation. Molecular docking simulations further illustrated interactions between virulence proteins and bioactive compounds derived from garlic and cumin essential oils, providing insight into their potential antifungal mechanisms.

**Table 3:** Effects of cuminoil on the powdery mildew in cucumber plants virulence protein using the docking analysis.

Protein\XYZ	Ligand name	Pubchem ID	Types of bond	Binding affinity score
Chitin_synthase (A0A068FR90) X = 10.666 Y = -19.105 Z = 3.927	Cyclohexene, 1-methyl-4-(1-methylethylidene)	92155	Carbon Hydrogen bond Conventional Hydrogen Bond Van der waals Pi-Alkyl Pi-Anion Alkyl	-8.4
	9,10-Anthracenedione, 1,4-diamino-2-methoxy-	17885		-7.8
	9b,10-tetrahydro-8-methoxy-5-methylindeno[1,2-b]indol	15082742		-7.2
	Aromadendrene	11095734		-6.6
	Caryophyllene oxide	1742210		-6.4
	Bicyclo[3,1,0]hepta-2-enomythyl-5-(1-methylethyl)	17868		-5.8

**Table 4:** Effects of garlic oil and ascorbic acid, fungicide (Topas^®^) (B) on the powdery mildew in cucumber plants virulence protein using the docking analysis.

Protein\XYZ	Ligand name	Pubchem ID	Types of bond	Binding affinity score
Chitin_synthase (A0A068FR90) X = 10.666 Y = -19.105 Z = 3.927	Cyclohexene,4-(4-ethylcyclohexyl)-1-pentyl-	543386	Alkyl Pi-Anion	-7.5
	1-Methyl-2-methylenecyclohexane	137725		-6.5
	Heptadecane	12398		-5.8
	Topas	91693		-7.9
	Ascorbic Acid	54670067		-6.6

### 3.11 Correlation study

**Figure 11:**
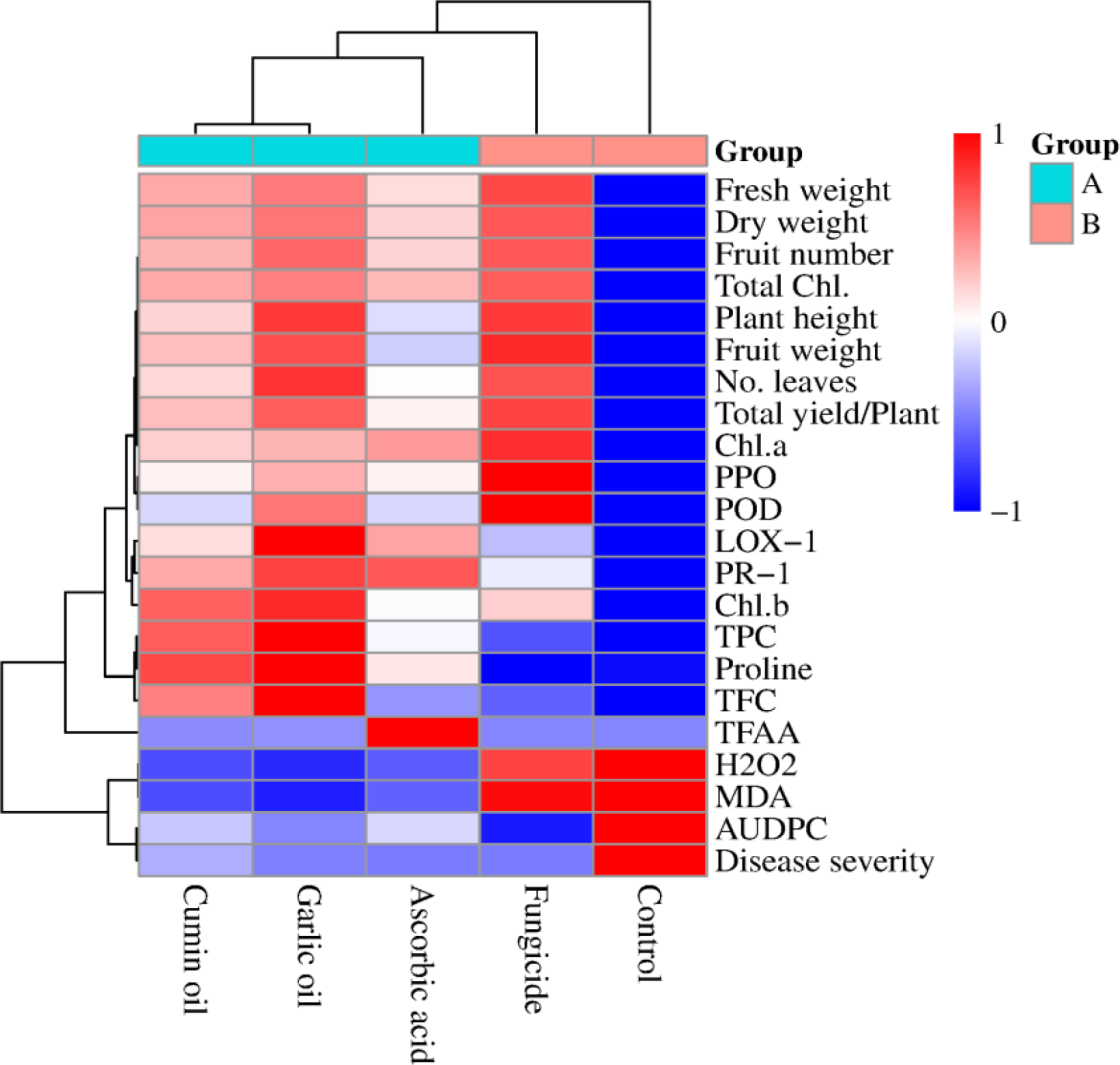
Cluster heatmap analysis shows of the agro-physiological and biochemical and genetic features of cucumber plants infested with powdery mildew and treated garlic oil, cumin oil, ascorbic acid, and fungicide, and control (untreated plant). Chl., chlorophyll; PPO, polyphenol oxidase; POD, peroxidase; TPC, total phenol content; TFC, total flavonoids content; TFAA, total free amino acids; H_2_O_2,_ Hydrogen peroxide content; MDA, malondialdehyde.

**Figure 12:**
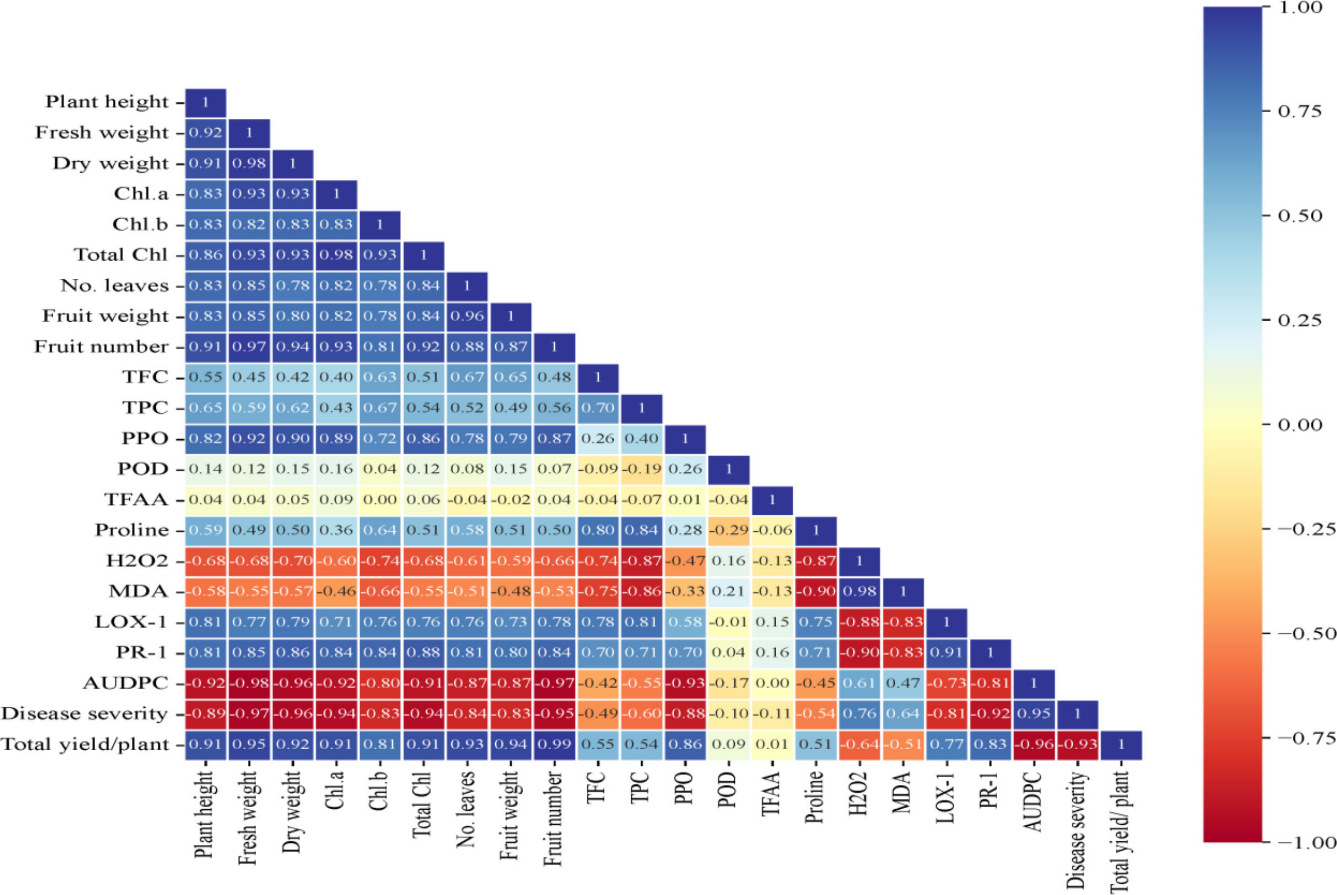
Matrix correlation analysis shows the correlation between the agro-physiological, biochemical and genetic properties of plants infested with powdery mildew and treated garlic oil, cumin oil, ascorbic acid, and chemical fungicide, and control (untreated plant). Chl., chlorophyll; PPO, polyphenol oxidase; POD, peroxidase; TPC, total phenol content; TFC, total flavonoids content; TFAA, total free amino acids; H_2_O_2,_Hydrogen peroxide content; MDA, malondialdehyde.

The analysis of the cluster heatmap and matrix correlation reveals changes in the agro-physiological, biochemical, and genetic traits of cucumber plants infested with powdery mildew and treated with garlic oil, cumin oil, ascorbic acid, and a fungicide (**Figures 11**, **12**). The cluster heatmap, which included 22 parameters, classified the treatments into two distinct clusters: Cluster A, consisting of cumin oil, garlic oil, and ascorbic acid, and Cluster B, which included untreated plants (control) and fungicide (**Figure 11**). The heatmap indicated that natural oil treatments, particularly garlic and cumin oils, exhibited a consistent response pattern characterized by improved agronomic properties, including plant height, number of leaves, plant fresh weight, plant dry weight, chlorophyll a (Chl.*a*), number of fruits, fruit weight, and total yield per plant. Additionally, these treatments had a positive effect on antioxidant-related parameters (PPO, POD, TFC), the accumulation of osmoprotectants (proline and total free amino acids, TFAA), and the relative expression of defense-associated genes (LOX-1 and PR-1). In contrast, oxidative stress markers, such as hydrogen peroxide (H_2_O_2_), malondialdehyde (MDA), and AUDPC values, were significantly reduced compared to the control. Furthermore, the cluster heatmap analysis indicates that ascorbic acid positively influences morphological traits and PR-1 expression. However, its efficacy is lower compared to the essential oils tested, specifically garlic and cumin oils. The control treatment exhibited the lowest cucumber growth parameters as well as the highest levels of oxidative stress markers (H_2_O_2_, MDA) and disease severity.

Likewise, the matrix correlation analysis was performed to examine the positive and negative relationships among the measured parameters (**Figure 12**). Positive correlations are indicated in blue, while negative correlations are shown in red, as illustrated in **Figure 12**. This analysis showed strong positive associations among all growth and yield traits, with plant height, fresh and dry weights, fruit number, and total yield per plant exhibiting correlations exceeding 0.90. Chlorophyll pigments (Chl.a, Chl.b, and total Chl.) demonstrated a strong correlation with growth attributes, indicating that increased photosynthetic activity is closely associated with improved plant performance. Furthermore, enzymatic and non-enzymatic antioxidants, including PPO, proline, TFC, and TPC, showed positive correlations with both growth traits and PR-1 expression. This finding confirms their coordinated role in activating defense mechanisms. Conversely, oxidative stress markers (H_2_O_2_ and MDA) were strongly negatively correlated with growth, pigment levels, yield, and defense enzymes (r = −0.80), highlighting the detrimental effects of stress accumulation. Disease severity and AUDPC exhibited the most significant negative correlations with all growth and physiological parameters (up to −0.95), underscoring their critical role in yield loss. Overall, the matrix reveals a clear pattern where enhanced defense responses and reduced oxidative stress are closely associated with improved plant health under powdery mildew infestation.

## 4 Discussion

This study provides integrated physiological, biochemical, molecular, and computational evidence supporting the effectiveness of natural essential oils, particularly garlic and cumin oils, as sustainable alternatives for controlling cucumber powdery mildew caused by *P. xanthii*. Powdery mildew infection significantly impaired cucumber plant health, as indicated by increased disease incidence and severity in untreated controls (**Figures 1**, **2**). Application of essential oils, especially garlic and cumin oils, effectively suppressed disease development, with garlic oil exhibiting the strongest inhibitory effect, followed by cumin oil and ascorbic acid. These findings are consistent with previous studies reporting that garlic extracts substantially reduce powdery mildew intensity (Elagamey et al., 2023), while other plant oils, including lemon, thyme, and lemongrass, also mitigate fungal infections in greenhouse cucumbers (Mostafa et al., 2021). The antifungal activity of these oils is largely attributed to bioactive compounds, including organosulfur molecules, cuminaldehyde, γ-terpinene, and terpenoids, which disrupt fungal cell walls, inhibit ergosterol biosynthesis, and compromise membrane integrity (Yingngam, 2022). Molecular docking analyzes further suggested potential interactions between these bioactive compounds (e.g., allicin, cuminaldehyde, and trans-caryophyllene) and the fungal pathogenicity-related protein CasA3, which is essential for cell wall biosynthesis and virulence (Hu et al., 2024; Azam et al., 2025; Dong et al. 2023). Molecular docking arises as an essential tool in agricultural innovation, allowing the investigation of bioactive compounds that improve disease control though decreasing dependence on chemical pesticides. By encouraging the rational use of natural molecules, docking donates not only to reducing environmental contamination but to advancing additional sustainable agricultural systems (Iñiguez-Luna et al., 2025) Molecular docking is a computational technique that predicts the binding affinity of ligands to receptor proteins. It has grown into a powerful tool for medication development, notwithstanding its possible use in nutraceutical research (Agu et al., 2023). These docking results should be regarded as hypothesis-generating, as they provide mechanistic insights rather than direct evidence of *in vivo* activity. When interpreted in conjunction with physiological data, they suggest that essential oils may exert their effects through a dual mode of action, combining direct antifungal activity with the induction of host defense responses. The target protein used in the docking analysis was selected based on its critical biological role in fungal growth and pathogenicity. In fungal pathogens, cell wall biosynthesis is an essential process required for maintaining structural integrity, facilitating host invasion, and sustaining disease development (Shree et al., 2024). *Podosphaera xanthii*, the main causal agent of powdery mildew in cucurbits, employs effector proteins that modify, degrade, or sequester immunogenic chitin oligomers, facilitating fungal pathogenesis and disease development. In addition, chitin synthase (CHS) is a key enzyme required for chitin biosynthesis and fungal cell wall formation, particularly during haustorial development. Therefore, inhibition of cell wall biosynthesis represents a promising antifungal strategy against *P. xanthii* (Bakhat et al., 2023). Therefore, targeting chitin synthase (CHS)proteins represents a biologically relevant and mechanistically justified approach for molecular docking studies. Inhibition of this enzyme is expected to impair cell wall formation, ultimately leading to reduced fungal growth and virulence. The current study further demonstrated that garlic oil effectively reduced disease severity and AUDPC values, showing a suppressive effect against *P. xanthii* that was statistically comparable to the chemical fungicide Topas.

Powdery mildew infection negatively affected vegetative growth and yield, as reflected by reductions in plant height, leaf number, chlorophyll content, fruit weight, and total yield (**Table 2**; **Figure 3**). The reduction in disease severity was closely associated with improvements in these growth and yield parameters. Treatments with garlic and cumin oils significantly alleviated the negative effects of the disease, likely by preserving leaf integrity, photosynthetic efficiency, and carbon assimilation. Garlic oil and the chemical fungicide exhibited the greatest increases in fruit number and weight, followed by cumin oil and ascorbic acid, suggesting a strong cause-and-effect relationship between disease suppression and enhanced productivity. Heatmap analyzes revealed strong positive correlations among vegetative growth and yield parameters, whereas disease severity was negatively correlated (**Figures 11**, **12**). Garlic oil resulted in the highest fruit weight and total yield, followed by cumin oil and ascorbic acid, indicating that effective disease suppression combined with enhanced metabolic activity supports reproductive success. Ascorbic acid induced moderate growth and yield improvements, likely through maintenance of physiological balance and cellular protection rather than direct antifungal activity (Boateng et al., 2024). In contrast, the chemical fungicide provides direct suppression of the pathogen by inhibiting key fungal metabolic and developmental processes, thereby limiting pathogen colonization and tissue damage (Savary et al., 2019). Effective disease control preserves leaf integrity and chloroplast structure, maintaining photosynthetic efficiency, chlorophyll content, and carbon assimilation, which collectively support greater biomass accumulation and overall plant growth. Essential oils and ascorbic acid, while comparably effective in promoting growth parameters, also activate plant defense mechanisms, representing environmentally friendly alternatives to synthetic fungicides (Abdelfatah et al., 2025). These observations align with previous reports demonstrating improved shoot length, leaf area, and fruit productivity in cucumbers under powdery mildew stress when treated with essential oils (Soleimani et al., 2024; Mostafa et al., 2021).

The treatment with essential oils, particularly garlic oil, significantly enhanced both enzymatic (e.g.,SOD, CAT, PPO, and POD) and non-enzymatic antioxidant activities (TPC, TFC, and proline), thereby alleviating oxidative stress caused by pathogen infection (Yang et al., 2021; Gan et al., 2025; Jain et al., 2025). Cumin oil also produced moderate antioxidant responses. Additionally, essential oil treatments facilitated the accumulation of TPC and TFC in cucumber leaves (**Figure 6**), which are essential for antimicrobial defense, ROS scavenging, and fortifying cell walls. Increased activities of PPO and POD likely utilize these phenolics to create lignin-like polymers and quinones, forming synergistic barriers against fungal invasion (Mayer, 2006; Mabrouk et al., 2025; Tiwari et al., 2025). Positive correlations among TPC/TFC, enzyme activities, and yield traits further highlight the functional importance of these metabolites under pathogen stress. Furthermore, osmoprotectants such as proline and TFAA accumulated following the application of essential oils (**Figure 6**). Proline serves multiple functions as an osmolyte, ROS scavenger, membrane stabilizer, and signaling molecule, while free amino acids act as precursors for pathogenesis-related (PR) proteins and secondary metabolites (Ghosh et al., 2022). Together, these biochemical adaptations create an integrated defense system that restricts the development of powdery mildew while supporting growth. Correlation analyzes revealed strong negative associations between TFC, TPC, and proline with MDA and H_2_O_2_ activities (**Figure 12**). Ascorbic acid conferred moderate tolerance to powdery mildew by enhancing antioxidant capacity, reducing lipid peroxidation, and partially restoring growth and yield (**Table 2**; **Figures 3**–**5**). As a crucial non-enzymatic antioxidant, ascorbic acid directly scavenges ROS and helps maintain redox homeostasis (Wu et al., 2024; Kaur et al., 2022). Additionally, it interacts with phenylpropanoid metabolism and stress-related signaling pathways, indirectly bolstering plant defense (Dixon and Paiva, 1995; Aluko et al., 2025). Although ascorbic acid is less effective than essential oils in directly suppressing disease, its protective effects on cellular integrity and metabolic balance render it a valuable supplementary component in integrated strategies for managing powdery mildew.

To clarify cucumber defense mechanisms against powdery mildew (*P. xanthii*), the expression of two biotic stress-responsive genes, linoleate 9S lipoxygenase 6-like (LOX-1) and pathogenesis-related 1 (PR-1) was quantified using real-time Polymerase Chain Reaction (PCR) Garlic and cumin oils further induced systemic resistance, evidenced by upregulation of defense-related genes PR-1 and LOX-1 (**Figure 7**). The results suggest that bioactive compounds in essential oils may act as elicitors of systemic resistance, enhancing host defense responses while potentially influencing pathogen interaction pathways. Overall, garlic oil consistently triggered the strongest transcriptional response, highlighting its potential as a potent inducer of plant immunity in integrated disease management strategies. In this contrast, PR-1 and LOX-1 genes are markers of salicylic acid (SA)-mediated systemic acquired resistance (Boamah et al., 2023). Concurrent induction of salicylic acid (SA)-mediated pathways suggests that essential oils activate multiple defense mechanisms, enhancing lignification, PR protein synthesis, and secondary metabolite accumulation. Plant PR1 proteins contribute to the host immune response, while pathogens’ PR1-like proteins are crucial to their pathogenicity due to their suppress the host’s immune responses and promote colonization (Han et al., 2023). Garlic oil elicited the strongest transcriptional response, correlating with superior disease suppression and yield improvements. Ascorbic acid induced moderate gene expression, consistent with its role in stress mitigation rather than direct defense elicitation. Overall, the integration of molecular, biochemical, and physiological responses confirms that essential oils function as both antifungal agents and elicitors of plant defenses, supporting their potential for sustainable and eco-friendly management of powdery mildew in cucumber (Nayak et al., 2024; Aroge et al., 2025).

## 5 Conclusion

The current study provides substantial evidence that garlic oil, cumin oil, and ascorbic acid effectively reduce the severity of powdery mildew (*P. xanthii*)in cucumbers, presenting eco-friendly alternatives to synthetic fungicides. Among these, garlic oil proved to be the most effective, significantly enhancing plant height, leaf number, biomass, chlorophyll content, fruit number, and fruit weight. Cumin oil followed in effectiveness, while ascorbic acid offered moderate benefits. Biochemically, the essential oils boosted antioxidant defenses by raising the levels of polyphenol oxidase (PPO) and peroxidase (POD), as well as the total phenolics, flavonoids, free amino acids, and proline. They also lowered oxidative stress markers like hydrogen peroxide (H_2_O_2_) and malondialdehyde (MDA). Garlic oil elicited the most robust responses. At the molecular level, garlic and cumin oils upregulated the genes PR-1 and LOX-1, activating pathways mediated by salicylic acid and jasmonic acid, which indicates systemic acquired resistance. Molecular docking revealed that Cyclohexene,4-(4-ethylcyclohexyl)-1-pentyl-Methyldiethylborane and Heptadecane from garlic essential oil had the highest docking scores with CesA3 protein, Additionally, cyclohexene, 9b,10-tetrahydro-8-methoxy-5-methylindeno,10-anthracenedione, trans-caryophyllene, bicyclo, and aromadendrene from cumin oil showed strong binding affinity with these protein. This suggests these compounds may inhibit fungal growth by targeting cellulose synthase, a key enzyme in cell wall formation. These findings demonstrate that garlic and cumin oils suppress powdery mildew through dual mechanisms: direct inhibition of the pathogen and activation of host defenses. Integrating these oils into sustainable integrated pest management (IPM) strategies could enhance cucumber productivity while decreasing reliance on chemical fungicides. Docking studies revealed that bioactive compounds, in essential oil bind to fungal pathogenicity proteins, thereby supporting their antifungal activity. Future research should prioritize field-scale validation, formulation development, and synergistic integration with resistant cultivars and sustainable agronomic practices to further minimize dependence on synthetic fungicides and promote environmentally resilient crop protection strategies.

## Data Availability

Dataset available on request from the authors.
