# Supplementary material for: Agro-physiological responses and molecular docking evaluation of some essential oils and ascorbic acid as eco-friendly inhibitors of powdery mildew in cucumber plants
Source: Front Plant Sci. 2026 Jun 12;17:1786347. doi: 10.3389/fpls.2026.1786347 (PMC13303492; doi:10.3389/fpls.2026.1786347)
Supplement: Supplementary file 1 [file Table1.docx]

Supplementary Material


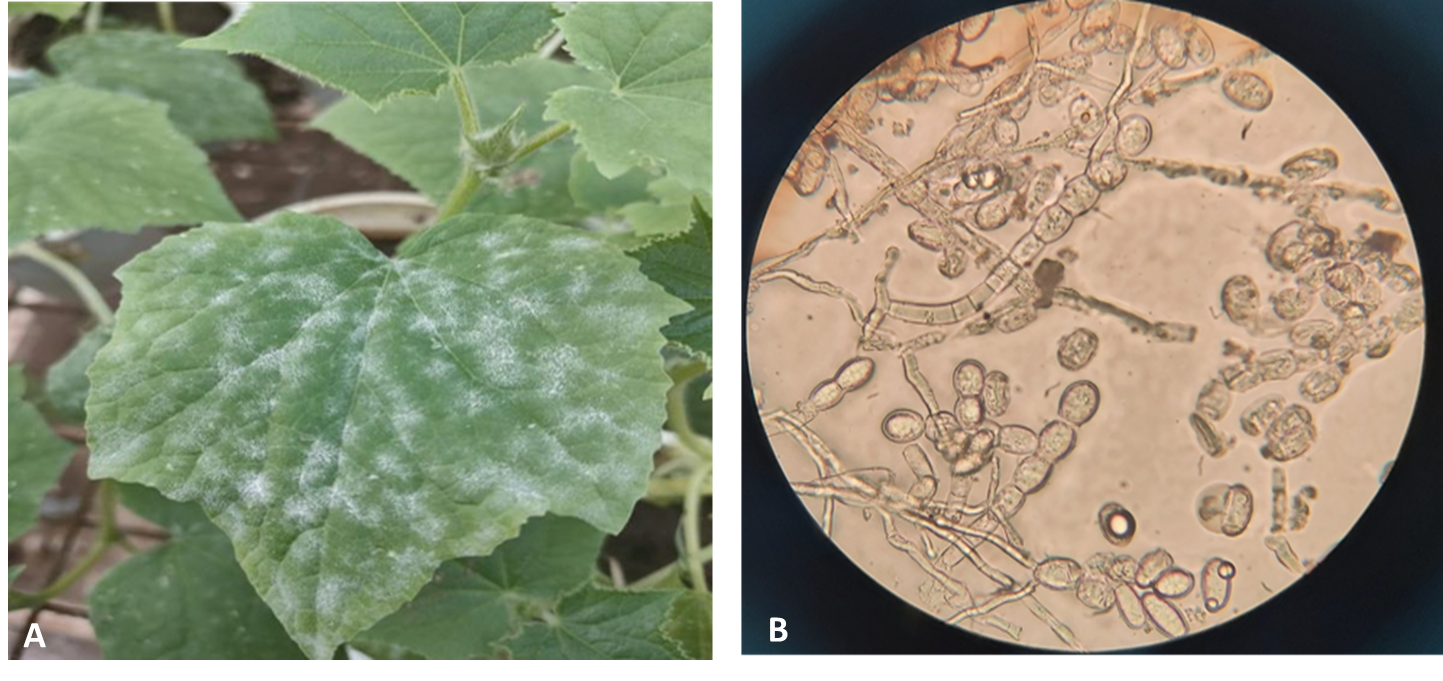


**Figure S1.** The characteristic symptoms of the pathogen, including white powdery patches (A) on the leaf surfaces and condia spores (B) of *P. xanthii .*

# Supplementary Tables

**Supplementary Table S1:** Chemical components of the essential oils of garlic (*Allium sativum L*) determined by GC-MS methods*.*

| Molecular formula | | Name of compound | RT(min) | Peak | |
| --- | --- | --- | --- | --- | --- |
| C_6_H_8_S_2_ | 3-Vinyl-1,2-dithiacyclohex-4-ene | | 6.645 | 1 | |
| C_21_H_44_ | n-Heneicosane | | 7.623 | 2 | |
| C_35_H_72_ | n-Pentatriacotane | | 8.864 | 3 | |
| C_36_H_74_ | n-Hexatriacontane | | 8.864 | 4 | |
| C_18_H_38_ | Octadecane | | 9.019 | 5 | |
| C_16_H_32_O_2_ | Hexadecanoic acid | | 11.205 | 6 | |
| C_25_H_48_ | Dodecane, 1-cyclopentyl-4-(3-cyclopentylpropyl)- | | 14.512 | 7 | |
| C_18_H_38_ | Heptadecane, 3-methyl- | | 14.615 | 8 | |
| C_15_H_32_ | Pentadecane | | 15.594 | 9 | |
| [C_15_H_30_](http://www.chemexper.com/cheminfo/servlet/org.chemcalc.ChemCalc?isograph=on&mformula=C15H30) | 1-pentadecene | | 17.402 | 10 | |
| C_15_H_28_ | 10.alpha.-Eremophilane | | 17.402 | 11 | |
| C_10_H_18_ | Bicyclo[3.1.1]heptane, 2,6,6-trimethyl- | | 17.436 | 13 | |
| Unknown | 2.2.6-trimethyl-biocyclo[4.1.0]hept-1-yl-methanol | | 18.832 | 14 | |
| C_16_H_34_ | Hexadecane | | 19.130 | 15 | |
| C_10_H_18_ | Trans-pinane -[Bicyclo[3.1.1]heptane, 2,6,6-trimethyl-](http://webbook.nist.gov/cgi/cbook.cgi?ID=C473552&Units=SI) | | 19.353 | 16 | |
| C_10_H_20_O | Cyclohexanol, 5-methyl-2-(1-methylethyl)- | | 20.841 | 18 | |
| C_14_H_30_ | Tridecane, 3-methyl- | | 20.841 | 19 | |
| C_13_H_28_ | decane,2,6,8-trimethyl- | | 20.841 | 20 | |
| C_17_H_34_ | Undecane, 2-cyclohexyl- | | 21.047 | 21 | |
| Unknown | Borinic asid, diethyl-,1-cyclododecen-1-yl ester | | 21.047 | 22 | |
| C_9_H_18_ | Cyclopentane, 1-methyl-3-(1-methylethyl)- | | 21.047 | 23 | |
| Unknown | Hahnfett | | 21.138 | 24 | |
| Unknown | Cycloundecane,(1-methylethyl)- | | 21.138 | 25 | |
| C_5_H_13_BO | Borane ,diethyl methyl- | | 21.350 | 26 | |
| C_8_H_14_ | 1-Methyl-2-methylenecyclohexane | | 21.899 | 27 | |
| C_10_H_18_ | Bicyclo[2.2.1]heptane, 2,2,3-trimethyl-, endo- | | 21.899 | 28 | |
| C_17_H_34_ | 1-Heptadecene | | 22.277 | 30 | |
| C_20_H_40_ | Cyclotetradecane, 1,7,11-trimethyl-4-(1-methylethyl)- | | 22.277 | | 31 |
| C_17_H_36_ | Heptadecane | | 22.649 | | 32 |
| C_19_H_40_ | 2,6,10,14-Tetramethylpentadecane | | 22.832 | | 33 |
| C_18_H_36_ | 1-Octadecene | | 23.645 | | 34 |
| C_19_H_38_ | Tridecane, 4-cyclohexyl- | | 24.640 | | 38 |
| C_18_H_36_ | Dodecane, 6-cyclohexyl- | | 24.812 | | 39 |
| C_18_H_32_O | Koiganal ll | | 24.943 | | 40 |
| C_18_H_38_ | Heptadecane, 3-methyl- | | 25.075 | | 41 |
| (CH_3_)B(C_2_H_5_)2 | Methyldiethylborane | | 25.075 | | 42 |
| C_16_H_26_O_3_ | 2-Dodecen-1-yl(-)succinic anhydride | | 25.212 | | 43 |
| [C_16_H_26_O_3_](http://www.chemexper.com/cheminfo/servlet/org.chemcalc.ChemCalc?isograph=on&mformula=C16H26O3) | 2,5-furandione,3-(dodecenyl)dihydro- | | 25.212 | | 44 |
| C_16_H_26_O_3_ | 2-Dodecen-1-yl(-)succinic anhydride | | 25.579 | | 45 |
| C_18_H_30_O | 5,9,13-Pentadecatrien-2-one, 6,10,14-trimethyl- | | 25.767 | | 46 |
| C_18_H_38_ | Octadecane | | 26.094 | | 47 |
| C_20_H_42_ | Hexadecane, 2,6,10,14-tetramethyl- | | 26.380 | | 48 |
| C_19_H_38_ | 1-Nonadecene | | 26.820 | | 49 |
| Unknown | cyclohexane, 1,2-dimethyl-3-phenyl-4-propyl- | | 27.015 | | 50 |
| Unknown | 5,9-dimethyl-4,10-heptadecadiene* | | 27.038 | | 51 |
| Unknown | Cyclohexene,4-(4-ethylcyclohexyl)-1-pentyl- | | 27.038 | | 52 |
| Unknown | 2,5-furandione,9-dodecyl-(cis)-2-nonadecene | | 27.152 | | 53 |
| C_20_H_40_ | 3-Eicosene, (e)- | | 27.324 | | 54 |

**Supplementary Table S2.** Chemical components of the essential oils of cumin (*Cuminum cyminum L*) determined by GC-MS methods*.*

| Molecular formula | Name of compound | RT(min) | Peak |
| --- | --- | --- | --- |
| C_10_ H_16_ | Bicyclo[3.1.0]hexa-2-ene,2-methyl-5-(1-methyleethyl) | 7.349 | 1 |
| C_10_ H_16_ | Alpha thujene | 7.349 | 2 |
| Unknown | Alpha –pinene | 7.560 | 3 |
| C_10_ H_16_ | Bicyclo[3.1.0]hept-2-ene, 2,6,6-trimethyl | 7.560 | 4 |
| C_11_ H_18_ O_2_ | Bicyclo[3,1,1]heptane,6,6,-dimethyl-2-methylen-eptan | 7.560 | 6 |
| C_10_H_16_ | Bicyclo[3,1,0]hepta-2-enomythyl-5-(1-methylethyl) | 7.560 | 7 |
| C_10_H_16_ | Beta-pinene6,6-dimethyl- 2-methylenebicyclo [3.1.1] heptane | 7.560 | 8 |
| C_10_H_16_ | Phellandrene  1,3-cyclohexadiene ,2-methyl-5-diene | 9.374 | 9 |
| C_10_H_16_ | 1,3-Cyclohexadiene, 1-methyl-4-(1-methylethyl) | 9.694 | 10 |
| C_10_H_14_ | Benzene  1-methyl-2-(1-methylethyl)- | 9.975 | 11 |
| C_10_H_14_ | 1-Isopropyl-4-methylbenzene; 4-Isopropyltoluene | 9.975 | 12 |
| C_10_H_16_ | Terpinene  4-methyl-1-(1-methylethyl)-1,3-cyclohexadiene | 10.833 | 13 |
| C_10_H_16_ | Terpinene  4-methylene-1-(1-methylethyl)cyclohexene | 10.925 | 14 |
| C_10_H_16_ | Cyclohexene, 1-methyl-4-(1-methylethylidene) | 11.531 | 15 |
| C_10_H_16_O | Cyclohexanone, 5-methyl-2-(1-methylethenyl)-, trans- | 13.574 | 16 |
| C_10_H_18_O | 3-cyclohexen-1-ol,4-methyl-1-(1-methylethyl) | 13.866 | 17 |
| C_10_ H_12_ O | 1,3,3-trimethylcyclohex-1-ene-4-carboxaldehyde, | 14.232 | 18 |
| C_10_H_14_O | Propanal,2-methyl-3-phenyl | 15.588 | 19 |
| C_10_H_12_O | Benzaldehyde, 4-(1-methylethyl)- | 15.685 | 20 |
| C_10_H_16_O | 1-Cyclohexene-1-carboxaldehyde, 4-(1-methylethyl)- | 16.252 | 21 |
| C_10_H_14_O | 1-phenyl-1-butanol | 16.532 | 22 |
| C_10_H_14_O | Alpha-n-propyl benzyl alcohol | 16.561 | 23 |
| [C_11_H_16_O](http://www.chemexper.com/cheminfo/servlet/org.chemcalc.ChemCalc?isograph=on&mformula=C11H16O) | 2,2-dimethyl-1-phenylpropan | 16.710 | 24 |
| C_8_H_10_O | Benzenemethanol, α-methyl- | 16.738 | 25 |
| C_10_H_16_O | 1,4-Cyclohexadiene-1-methanol, 4-(1-methylethyl)- | 17.637 | 26 |
| C_15_H_24_ | Valencene  Naphthalene, 1,2,3,5,6,7,8,8a-dimethyl 1-7-(methylethyenyl)- | 18.438 | 27 |
| C_15_H_24_ | Trans-caryophyllene  bicyclo[7.2.0]undec-4-ene 4,11,11-trimethyl-8-methylene- | 19.347 | 28 |
| C_15_ H_24_ | bicyclo[3.1.1] hepta -2-ene,2,6-dimethyl-6-(4-methyl-3-pentenyl) | 19.611 | 29 |
| C_15_H_24_ | 1,6,10-Dodecatriene, 7,11-dimethyl-3-methylene | 20.017 | 30 |
| C_15_H_24_ | 2H-2,4a-methanonaphthalene, 1,3,4,5,6,7-hexahydro-1,1,5,5-tetramethyl | 20.446 | 31 |
| C_15_H_24_ | Zingiberene 1,3-Cyclohexadiene, 5-(1,5-dimethyl-4-hexenyl)-2-methyl- | 20.503 | 32 |
| C_15_H_24_ | [Aromadendrene](http://webbook.nist.gov/cgi/cbook.cgi?ID=C109119917&Units=SI) | 21.035 | 33 |
| C_15_H_24_ | Beta-bisabolene yclohexene, 1-methyl-4-(5-methyl-1-methylene-4-hexenyl) | 21.127 | 34 |
| C_15_H_24_O | henol, 2,6-bis(1,1-dimethylethyl)-4-methyl | 21.253 | 35 |
| C_15_H_24_O | Caryophyllene oxide | 22.781 | 36 |
| C_16_H_34_ | Hexadecane | 22.815 | 37 |
| C_15_H_26_O | Carotol  3a(1H)-Azulenol,2,3,4,5,8,8a-hexahydro-6,8a-dimethyl-3-(1-methylethyl)- | 23.067 | 38 |
| C_12_H_14_O_4_ | 1,3-Benzodioxole, 4,5-dimethoxy-6-(2-propenyl)- | 23.581 | 39 |
| C_15_H_26_O | Τ-muurolol-Naphthalenol, 1,2,3,4,4a,7,8,8a-octahydro-1,6-  dimethyl-4-(1-methylethyl)-, [1r-(1α,4β,4aβ,8aβ)]- | 24.446 | 40 |
| C_21_H_40_O_2_ | Octadecyl acrylate | 24.623 | 41 |
| C_17_H_36_ | Heptadecane | 24.669 | 42 |
| C_15_H_24_ | Cyclohexene, 1-methyl-4-(5-methyl-1-methylene-4-hexenyl) | 26.157 | 43 |
| C_18_H_38_ | Octadecane | 24.460 | 44 |
| C_19_H_40_ | 2,6,10,14-Tetramethylpentadecane | 24.817 | 45 |
| C_54_H_108_Br_2_ | tetrapentacontane,1,54-dibromo- | 25.996 | 46 |
| C_13_H_20_O | 3-Buten-2-one, 4-(2,6,6-trimethyl-2-cyclohexen-1-yl)- | 27.192 | 47 |
| C_18_H_36_O | 2-Pentadecanone, 6,10,14-trimethyl- | 27.307 | 48 |
| C_18_H_36_ | Dodecylcyclohexane | 27.678 | 49 |
| C_19_H_40_ | Nonadecane | 28.159 | 50 |
| C_16_H_32_O_2_ | Hexadecanoic acid | 29.515 | 51 |
| C_16_H_22_O_4_ | Dibutyl phthalate | 29.532 | 52 |
| C_20_H_42_ | Eicosane | 29.784 | 53 |
| C_21_H_44_ | n-Heneicosane | 31.341 | 54 |
| C_18_H_34_O_2_ | 9-Octadecenoic acid | 32.313 | 55 |
| C_18_H_36_O_2_ | Octadecanoic acid | 32.559 | 56 |
| C_15_H_12_N_2_O_3_ | 9,10-Anthracenedione, 1,4-diamino-2-methoxy- | 33.784 | 57 |
| C_23_H_48_ | n-Tricosane | 34.265 | 58 |
| Unknown | 9b,10-tetrahydro-8-methoxy-5-methylindeno[1,2-b]indol | 34.619 | 59 |
| Unknown | 2-phenyl-4,7dimethyl-5-oxipyrano[4,3-b]pyridine | 34.619 | 60 |
| C_26_H_52_ | Eicosane, 9-cyclohexyl- | 34.871 | 61 |
| C_6_H_11_Br | Cyclohexane, bromo | 38.115 | 62 |

**
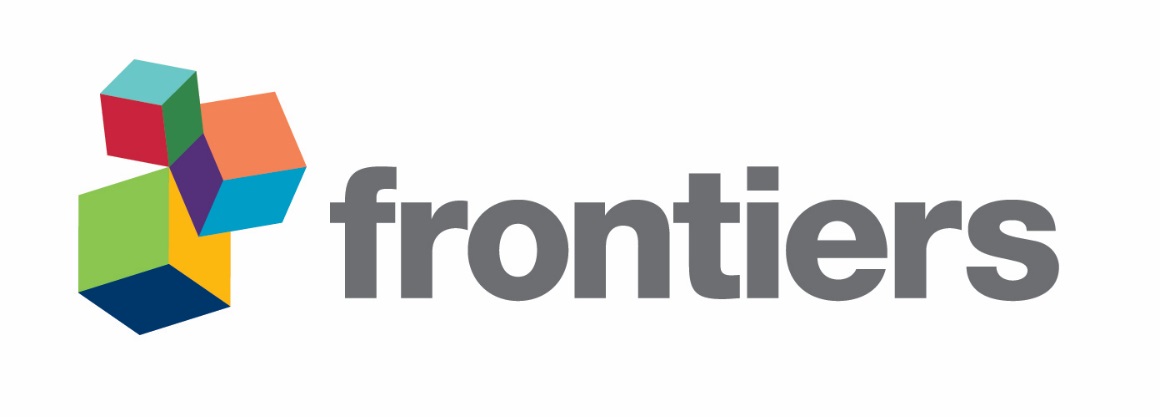
**

**Supplementary Figure 1.** The figure legends are required to have the same font as the main text, 12 point normal Times New Roman, single spaced. Please use a single paragraph for each legend and prepare the figures keeping in mind the PDF layout.
